# Identification and Validation of Novel Chromosomal Integration and Expression Loci in *Escherichia coli* Flagellar Region 1

**DOI:** 10.1371/journal.pone.0123007

**Published:** 2015-03-27

**Authors:** Mario Juhas, James W. Ajioka

**Affiliations:** Department of Pathology, University of Cambridge, Cambridge, United Kingdom; Imperial College London, UNITED KINGDOM

## Abstract

*Escherichia coli* is used as a chassis for a number of Synthetic Biology applications. The lack of suitable chromosomal integration and expression loci is among the main hurdles of the *E*. *coli* engineering efforts. We identified and validated chromosomal integration and expression target sites within *E*. *coli* K12 MG1655 flagellar region 1. We analyzed five open reading frames of the flagellar region 1, *flgA*, *flgF*, *flgG*, *flgI*, and *flgJ*, that are well-conserved among commonly-used *E*. *coli* strains, such as MG1655, W3110, DH10B and BL21-DE3. The efficiency of the integration into the *E*. *coli* chromosome and the expression of the introduced genetic circuit at the investigated loci varied significantly. The integrations did not have a negative impact on growth; however, they completely abolished motility. From the investigated *E*. *coli* K12 MG1655 flagellar region 1, *flgA* and *flgG* are the most suitable chromosomal integration and expression loci.

## Introduction

*Escherichia coli* K-12 is a model Gram-negative bacterium used as an intermediate and final destination chassis for a number of biotechnological applications and Synthetic Biology devices [1–5]. Integration and expression of genetic circuits from the *E*. *coli* chromosome is preferable to their introduction into the genome on plasmids due to the lower metabolic burden and no need for selective pressure [6]. Chromosomal integration strategies differ in their speed and flexibility. Recently developed methods include ‘the one-step clonetegration’ that relies on the bacteriophage integrase-mediated site-specific recombination between phage attachment (*att*) sites [7]. Besides transposons, phages and phage-derived elements [8, 9], Red recombinase of the bacteriophage λ [10] is often used to engineer *E*. *coli*. The recently developed Red recombinase-based strategies include those based on the yeast mitochondrial homing endonuclease I-SceI [9], knock-in/knock-out (KIKO) vectors [11], and plasmid pSB1K3(FRTK) [12].

The lack of suitable chromosomal integration loci is among the main hurdles of the current *E*. *coli* engineering efforts. Traditionally phage attachment (*att*) sites have been used as integration targets [7]; however, *att* sequences are not present in all commonly-used *E*. *coli* strains. Integration target sites should be well-conserved, non-essential, well-characterized and highly expressed. Previously we have analyzed four genes of the *E*. *coli* K12 MG1655 flagellar gene region 3a and identified one locus that fulfills all these criteria and supports high efficiency integration [12]. The identification and characterization of the alternative integration sites is crucial for the development of a robust synthetic biology toolkit. Some applications require integrations of a number of genetic circuits into the chromosome (f.i. measuring synergistic effect of mutltiple genetic circuits on the phenotype or integration of the repressor and other genetic circuit(s) regulated by this repressor). Thus identification and validation of other suitable chromosomal integration and expression target sites would significantly help progress. Here we analyse open reading frames of the *E*. *coli* flagellar region 1. This region of the *E*. *coli* chromosome shows high probability of being occupied by RNA polymerase [13, 14] and is therefore potentially suitable for integration and expression of genetic circuits. We integrated a genetic circuit harbouring the thermosensitive λ repressor [15] into five well-conserved open reading frames of the flagellar region 1. We propose *flgA* and *flgG* as the most suitable chromosomal integration and expression loci of the *E*. *coli* flagellar region 1.

## Materials and Methods

### Bacterial strains, plasmids, and growth conditions

Bacterial strains and plasmids used in this analysis are listed in Table 1. *Escherichia coli* strains were routinely grown in Luria-Bertani (LB) medium. When needed, LB broth was supplemented with kanamycin (50 μg/ml) or ampicillin (100 μg/ml). Liquid cultures were grown in LB broth on a rotatory shaker at 200 r.p.m. Solid cultures were grown on agar plates for 24 hours. Depending on the requirements, both liquid and solid cultures were grown at 30°C, 37°C or 42°C.

**Table 1:** Bacterial strains and plasmids used in this analysis.

	Characteristics	Reference
**Strains**		
K12 MG1655	*E*. *coli* wild type	[32]
Ec:flgAi	*E*. *coli* K12 MG1655 with integration in *flgA*	This study
Ec:flgFi	*E*. *coli* K12 MG1655 with integration in *flgF*	This study
Ec:flgGi	*E*. *coli* K12 MG1655 with integration in *flgG*	This study
Ec:flgIi	*E*. *coli* K12 MG1655 with integration in *flgI*	This study
Ec:flgJi	*E*. *coli* K12 MG1655 with integration in *flgJ*	This study
**Plasmids**		
pCP20	FLP recombinase helper plasmid	[10]
pKM208	Red recombinase controlled by lacZ	[33]
pSB1A1(GFP)	Amp^R^, GFP under the control of λ promoter	[12]
pSB1K3(FRTKr)	Kan^R^, λ repressor	[12]

### Recombinant DNA methodology

PCR amplifications of DNA fragments were performed using Dream Taq master mix kit (Thermo Scientific) or Phusion DNA polymerase (Thermo Scientific) in 50 μl volumes according to the supplier’s instructions. Oligonucleotide primers used in this analysis were synthesized by Integrated DNA Technologies (IDT). Plasmid isolations and gel extractions were carried out using Qiaprep Spin Miniprep kit (Qiagen) and Qiaquick Gel Extraction kit (Qiagen), respectively, according to the manufacturer’s instructions. Sequencing of DNA fragments was performed by Source Bioscience (Cambridge, UK). DNA fragments were assembled using the modified Gibson Isothermal Assembly method [16] as described previously (http://www.srcf.ucam.org/~wac26/gibson/index.html) [12]. The modified Gibson Isothermal Assembly reaction was incubated at 50°C for 60 min prior to *E*. *coli* transformation.

### *E*. *coli* chromosomal integration

Electro-competent and chemically-competent *E*. *coli* were prepared by the modified Miller and Nickoloff [17] and Hannah methods [18], respectively. Integrations into the *E*. *coli* chromosome were performed by method described previously [12]. Briefly, DNA fragment composed of the thermosensitive λ repressor construct, kanamycin and FRT sites flanked by sequences homologous to the *E*. *coli* chromosome target genes was amplified by PCR and gel-purified. *E*. *coli* strain K12 MG1655 was transformed with pKM208 harboring resistance to ampicillin and selected on ampicillin plates at 30°C. Overnight culture (1:100 dilution) of *E*. *coli* strain K12 MG1655 with pKM208 was inoculated into LB with ampicillin and grown at 30°C to OD_600_ of 0.2. 1 mM IPTG was added at this time point and the bacterial culture was grown to OD_600_ of 0.4–0.6. Cells were washed twice with 10% (v/v) glycerol and resuspended in 100 μl of 10% glycerol per 100 ml of starting culture. Purified DNA fragment was introduced into electro-competent cells by electroporation on a Bio-Rad micropulser. Transformants were selected on kanamycin plates at 37°C. Temperature sensitive pKM208 was cured out by incubation at 42°C. Successful integration into the *E*. *coli* was confirmed by PCR using the flanking primers.

### Quantitative measurement of GFP fluorescence

*E*. *coli* cultures were grown overnight and normalized to OD_600_ of 0.05. 200 μl of these were transferred into flat-bottomed black 96 well plates (Greiner BioOne, UK). The plates were incubated in a Fluostar Omega fluorimeter (BMG Labtech, UK) at 30°C for 3 hours followed by 10 hours at 42°C for the measurement of the GFP fluorescence with an automatically repeated protocol. Excitation filter 485–12, emission filter EM520, gain 1400, double orbital shaking at 200 rpm, and cycle time 60 min were used for the GFP fluorescence measurement.

### Plate reader measurement of the growth rate

The overnight *E*. *coli* cultures were normalized to OD_600_ of 0.05. The total volume of 200 μl was transferred into flat-bottomed clear 96 well plates (Sterilin Sero-Well, UK). The plates were incubated in a microplate reader (Fluostar Omega) (BMG Labtech) for 24 hours at 37°C and 30°C with an automatically repeated protocol. The following parameters were used for the absorbance measurement (600 nm absorbance filter, cycle time 60 min, double orbital shaking at 500 rpm).

### RNA isolation and RT-PCR

Isolate II RNA Mini Kit (Bioline) was used to isolate the total RNA from 10^9^ *E*. *coli* cells grown into mid-exponential phase (OD_600_ = 0.7). RNA was eluted with 60 μl of RNAse-free H_2_O and purified from genomic DNA contamination using a TURBO DNA-free Kit (Applied Biosystems) according to supplier’s instructions. cDNA was synthesized from 1 μg of purified total RNA using SuperScript III Reverse Transcriptase (Invitrogen) according to supplier’s instructions. QuantiTect SYBR Green PCR Kit (Qiagen), 7500 Fast Real-Time PCR System (Applied Biosystems) and MicroAmp Fast Optical 96-Well Reaction Plates (Applied Biosystems) were used to measure the expression levels of the target DNA sequences (100–150 bp long). RT-PCR primers were designed usig Primer3 Software. The following program was used to amplify target DNA sequences in the 7500 Fast Real-Time PCR System: initial activation (15 min, 95°C), followed by 35–45 cycles of denaturation (15 s 94°C), annealing (30 s 50–60°C) and extension (30 s 72°C). The relative expression was quantified with REST9 Software (Qiagen) employing Pfaffl method [19]. The experiments were carried out in triplicate and the means and standard errors were calculated.

### Motility assay

Motility assays were performed on motility agar plates. 13 cm motility agar plates composed of 100 ml motility agar (5 g NaCl, 10 g tryptone, 0.25% Bacto-Agar (Difco)) were let to set overnight and pre-warmed at 37°C before inoculation of bacteria. Overnight bacterial cultures (2 μl of the OD600 of 1.0) were spotted into the middle of the motility plates and incubated for 4–6 hours at 37°C.

### Sequence analyses and databases

The target flagellar genes sequences were obtained from the *E*. *coli* K-12 project website (http://www.xbase.ac.uk/genome/escherichia-coli-str-k-12-substr-mg1655). The National Center for Biotechnology Information (NCBI) website's (http://ncbi.nlm.nih.gov) BLASTN and TBLASTX [20] algorithms were used to search for similarities between DNA sequences.

## Results and Discussion

### Flagellar region 1 as target for chromosomal integration

**Fig 1:**
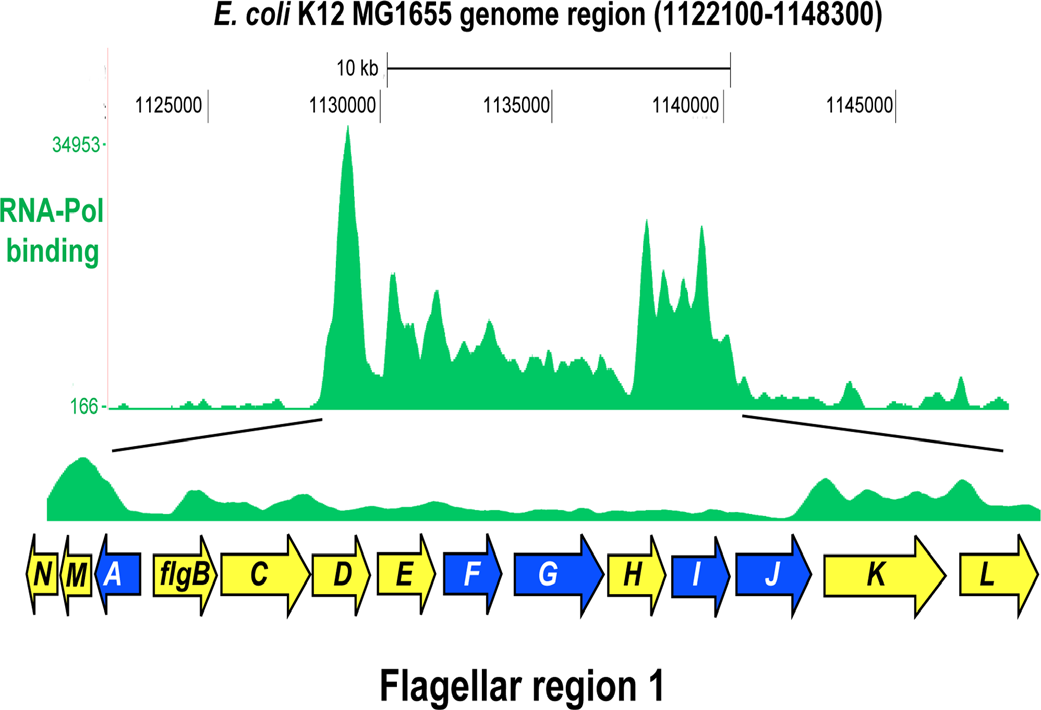
RNA polymerase binding to the *E*. *coli* flagellar region 1. Figure shows the ChIP-seq data of the binding of RNA polymerase (RNA-Pol) to the *E*. *coli* K12 MG1655 genome region (1122100–1148300). Flagellar region 1 (1128637–1140986) is enlarged to show location of its 14 open reading frames. The investigated integration target sites (*flgA*, *flgF*, *flgG*, *flgI* and *flgJ*) are highlighted blue. Figure was produced by uploading the RNA-Pol binding data [14] from cells at mid-exponential phase to the UCSC microbial genome browser for *E*. *coli* K12 MG1655 (http://microbes.ucsc.edu/cgi-bin/hgGateway?db=eschColi_K12). RNA-Pol binding is indicated by green peaks.

The main hurdle of *E*. *coli* engineering is the shortage of suitable loci that support high efficiency integration and expression of integrated genetic circuits [12]. Previously we showed that *fliT* is the most suitable integration site from the four analysed open reading frames of the *E*. *coli* K12 MG1655 flagellar region 3a [12]. The identification and validation of the alternative integration sites is crucial, particularly for applications requiring integrations of multiple genetic circuits into the chromosome. Here we investigate flagellar region 1 as potential target for chromosomal integrations. ChIP-seq data [13, 14] indicate high probability of the flagellar region 1 being occupied by RNA polymerase (Fig. 1), suggesting that genetic circuits integrated here would be highly expressed. Chromosomal integration sites should be well-conserved. BLAST search revealed that out of the 14 open reading frames of the *E*. *coli* K12 MG1655 flagellar gene region 1 (*flgA-L*), nine (*flgB*, *C*, *D*, *E*, *H*, *K*, *L*, *M*, *N*) were either missing or only partially conserved in some *E*. *coli* strains. The remaining five genes (*flgA*, *F*, *G*, *I*, *J*) are well-conserved among commonly-used *E*. *coli* strains, including MG1655, W3110, DH10B and BL21-DE3. *flgA* and *flgI* encode proteins for the flagellar basal-body periplasmic P ring assembly [21–24]. *flgF* and *flgG* encode flagellar components of the cell-proximal and the cell-distal part of basal body, respectively [25]. *flgJ* encodes *B*-N-acetylglucosaminidase that is essential for the bacterial flagella biogenesis [26]. The location of *flgA*, *flgF*, *flgG*, *flgI*, and *flgJ* in the *E*. *coli* K12 MG1655 flagellar region 1 are shown in Fig. 1. We used our streamlined Red recombinase-based chromosomal integration protocol [12] to integrate a genetic circuit (Repr-ts-1) consisting of the thermosensitive λ repressor expressed using a strong constitutive promoter, a RBS and a terminator taken from the iGEM Parts Registry into the investigated target genes *flgA*, *flgF*, *flgG*, *flgI*, and *flgJ* of the *E*. *coli* flagellar region 1.

### Chromosomal integrations into *flgA* and *flgG* occur with the highest frequency

**Fig 2:**
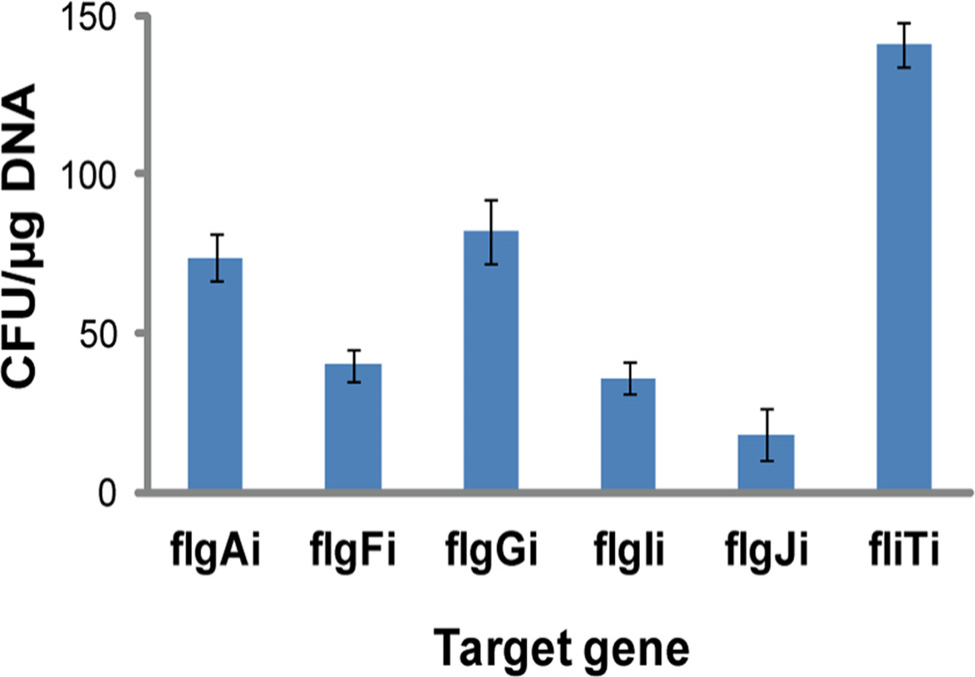
Efficiency of integration into the *E*. *coli* flagellar region 1. Figure shows the efficiency of integration into the target loci of the *E*. *coli* flagellar region 1 (*flgA* (flgAi), *flgF* (flgFi), *flgG* (flgGi), *flgI* (flgIi), and *flgJ* (flgJi)) calculated from the number of colony forming units (CFU) per μg of electroporated DNA. fliTi (integration efficiency of the best integration target locus of the flagellar region 3a [12]). The bars and errors represent averages and standard deviations calculated from three independent replicates.

Chromosomal integration efficiency differs significantly between integration sites, suggesting that to find the most suitable loci, the integration efficiencies have to be determined experimentally [12]. Genetic circuit Repr-ts-1 was integrated into five targeted open reading frames of the *E*. *coli* flagellar region 1: *flgA* (flgAi), *flgF* (flgFi), *flgG* (flgGi), *flgI* (flgIi), and *flgJ* (flgJi). Integration into the chromosome was verified by diagnostic PCR with flanking primers (S1 Fig.) and DNA sequencing. Primer sequences homologous to the target genes that were used to amplify Repr-ts-1 and the diagnostic flanking primers are listed in S1 Table. The integration efficiencies for the five targeted integration loci differed significantly. The integration of Repr-ts-1 into *flgA* and *flgG* occured with the highest efficiency (Fig. 2). The integrations into both *flgA* and *flgG* generated two times and four times more recombinants per μg DNA than integrations into *flgF/ flgI*, and *flgJ*, respectively. As *flgF*, *flgG*, *flgI*, and *flgJ* are part of one operon, while *flgA* is located in another operon, the observed differences in the integration efficiencies are not operon-specific. Notably, integrations into the analyzed genes of the *E*. *coli* flagellar region 1 occured with the lower efficiency than integrations into the best locus of the previously analyzed region 3a, *fliT* [12]. Integrations into *fliT* yielded approximately twice the number of recombinants than integrations into *flgA* and *flgG* (Fig. 2).

### Effect of integration into *flgA*, *flgF*, *flgG*, *flgI* and *flgJ* on motility and cell growth

**Fig 3:**
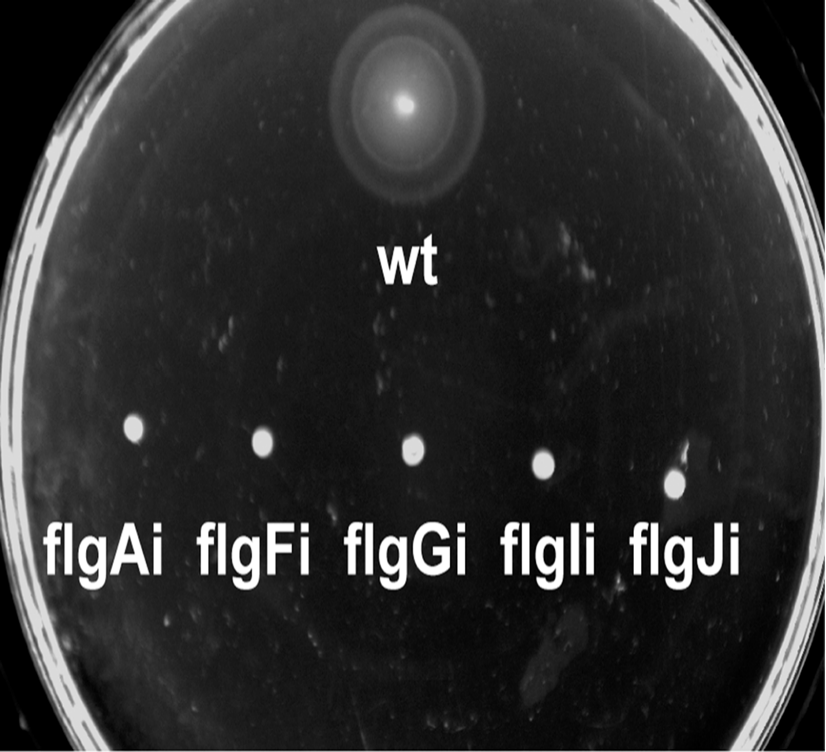
Motility Assay. Figure shows motility of the *E*. *coli* K12 MG1655 wild type (wt) and strains harboring integrations in the target loci of the flagellar region 1 (*flgA* (flgAi), *flgF* (flgFi), *flgG* (flgGi), *flgI* (flgIi), and *flgJ* (flgJi)). 2 μl of the overnight *E*. *coli* cultures (OD_600_ of 1.0) were inoculated in the middle of the motility plates and incubated for 4–6 hours at 37°C.

**Fig 4:**
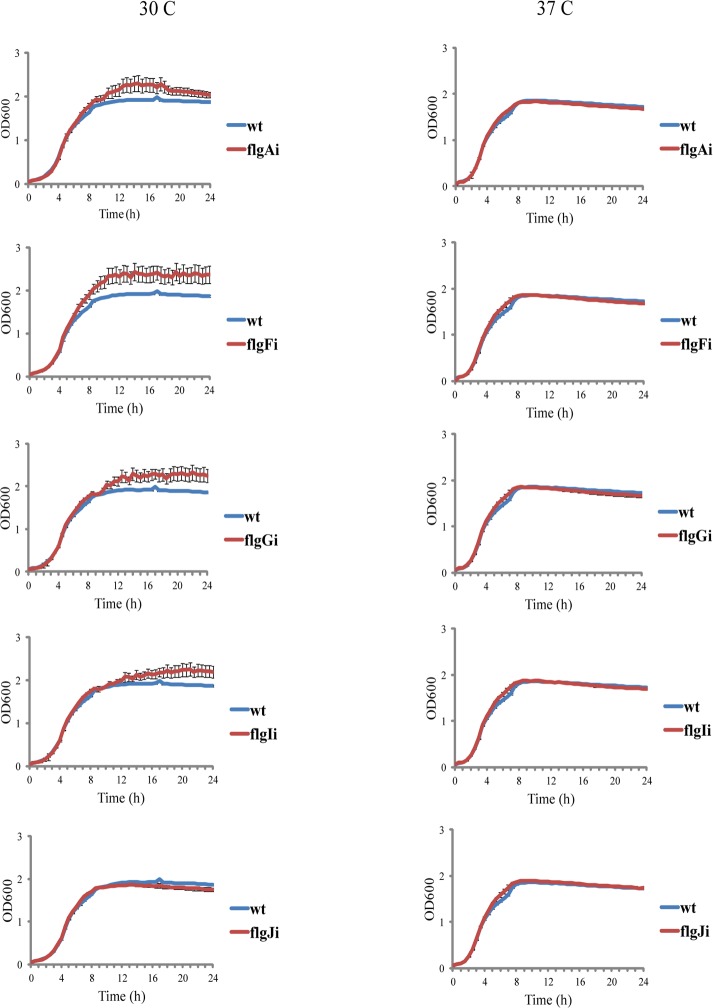
Growth rate of strains with integrations in the *E*. *coli* flagellar region 1. Figure shows growth curves of the *E*. *coli* K12 MG1655 wild type (wt) and strains harboring integrations in the target genes of the flagellar region 1 (*flgA* (flgAi), *flgF* (flgFi), *flgG* (flgGi), *flgI* (flgIi), and *flgJ* (flgJi)). Values represent the means calculated from three independent experiments. Raw plate reader data are displayed in the S2 Table.

Integration of the genetic circuit into the five target loci of the flagellar region 1 (flgAi, flgFi, flgGi, flgIi, and flgJi) completely abolished the motility of the engineered strains (Fig. 3). This is in contrast with the previously analysed flagellar region 3a where integrations into two of the four analysed loci had only a minor effect on motility [12]. As integrations should not inhibit growth, the target loci can not be located within essential genes [1, 5, 27–29]. The growth rates of the engineered strains harboring Repr-ts-1 integrations (flgAi, flgFi, flgGi, flgIi, and flgJi) and *E*. *coli* K12 MG1655 wild type were measured with the microplate reader (Fluostar Omega). Integrations into all five target loci did not have a negative impact on the growth at both the permissive (30°C) and the restrictive (37°C) conditions for the Repr-ts-1-bourne thermosensitive repressor (Fig. 4). Some of the engineered strains (flgAi, flgFi, flgGi, flgIi) grew to a higher final density than the wild type strain at permissive temperatures, while the growth of flgJi and the wild type strain was similar at both conditions (Fig. 4). This result is similar to that obtained for flagellar region 3a where strains with integrations grew to a higher density than the wild type strain [12]. The abolished motility and good growth (particularly of flgAi, flgFi, flgGi, flgIi) confirm the suitability of the tested loci for chromosomal integrations.

### Expression from *flgA*, *flgF*, *flgG*, *flgI* and *flgJ* of the flagellar region 1

**Fig 5:**
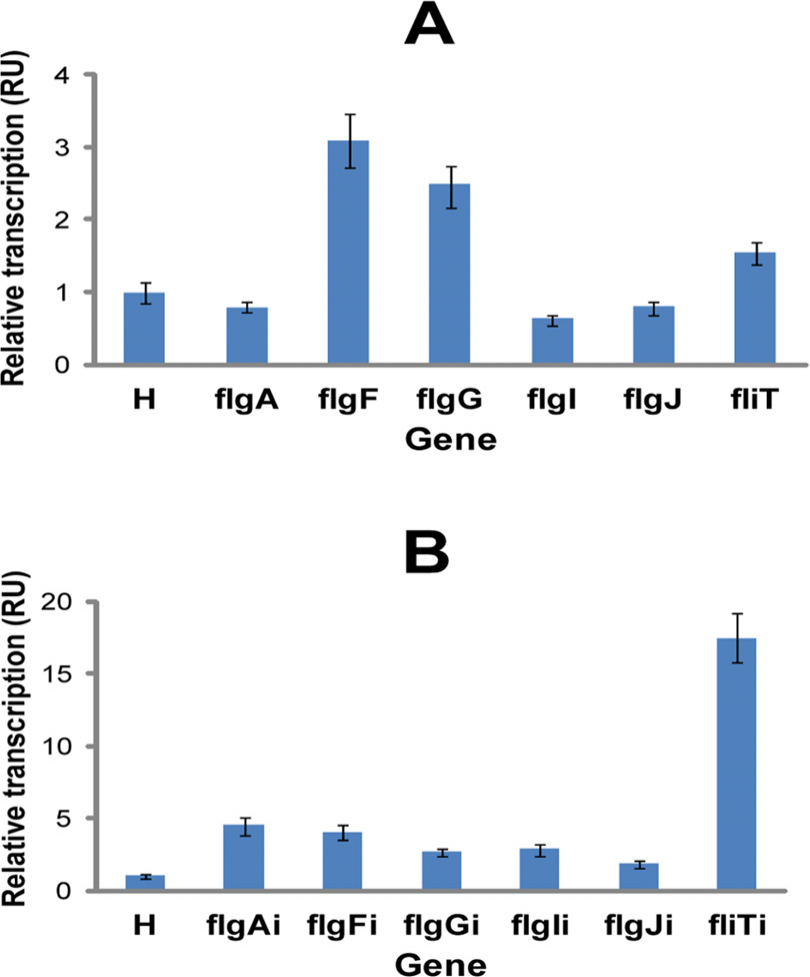
RT-PCR expression analysis of the target genes of the *E*. *coli* flagellar region 1. **(A)** RT-PCR-determined relative transcription of *flgA*, *flgF*, *flgG*, *flgI* and *flgJ* compared to the mean transcription of the house-keeping genes *arcA* and *rpoD* (H). **(B)** RT-PCR-determined relative transcription of the genetic circuit integrated into target loci of the *E*. *coli* flagellar region 1 (*flgA* (flgAi), *flgF* (flgFi), *flgG* (flgGi), *flgI* (flgIi), and *flgJ* (flgJi)) compared to the mean transcription of the house-keeping genes *arcA* and *rpoD* (H). fliT and fliTi (expression efficiency of the best integration target locus of the flagellar region 3a [12]). Transcription of each gene was assayed in triplicate and the mean was calculated. Error bars represent standard errors. RU (relative units of transcription quantified with REST9 Software (Qiagen) employing Pfaffl method [19]) (*flgA*, RU = 0.787, p = 0.053; *flgF*, RU = 3.063, p = 0.000; *flgG*, RU = 2.494, p = 0.000; *flgI*, RU = 0.626, p = 0.033; *flgJ*, RU = 0.807, p = 0.000; flgAi, RU = 4.499, p = 0.020; flgFi, RU = 4.004, p = 0.000; flgGi, RU = 2.697, p = 0.000; flgIi, RU = 2.894, p = 0.000; flgJi, RU = 1.854, p = 0.000).

**Fig 6:**
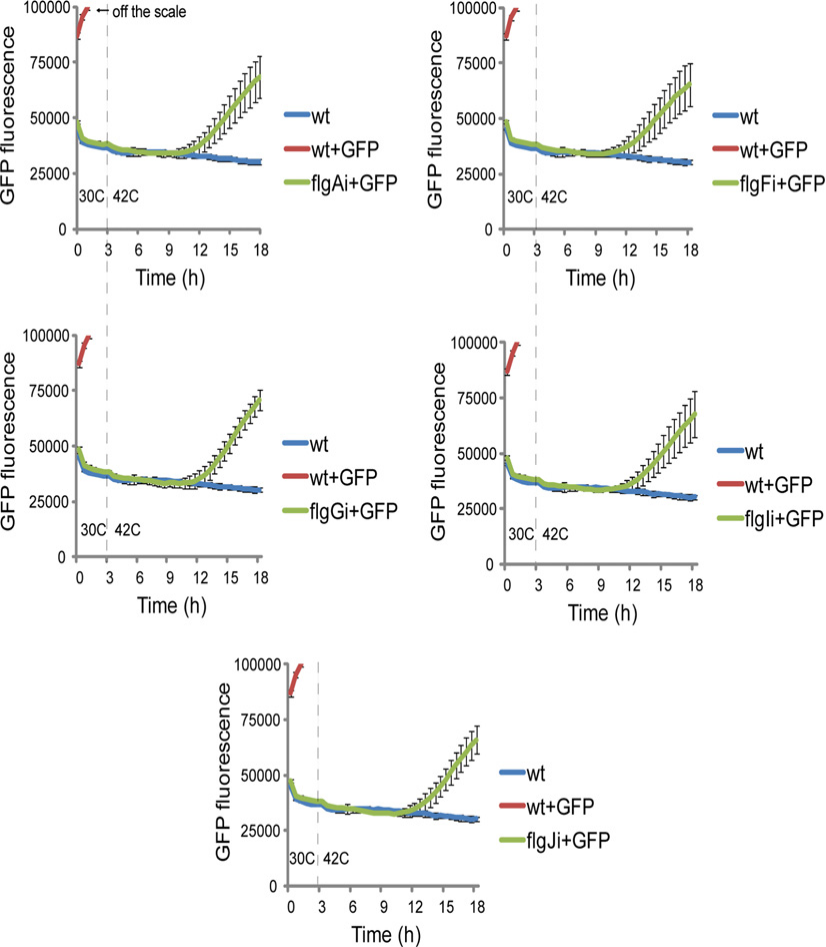
GFP fluorescence quantification. Verification of the chromosomal integration of the genetic circuit Repr-ts-1 by quantitative measurement of the GFP fluorescence over time with Fluostar Omega fluorimeter. Following temperature shift to 42°C after 3 hours (grey dashed line), the strains harboring integrations of the genetic circuit Repr-ts-1 in the target gene of the *E*. *coli* flagellar region 1 expressed GFP (*flgA* (flgAi), *flgF* (flgFi), *flgG* (flgGi), *flgI* (flgIi), and *flgJ* (flgJi) + GFP). GFP fluorescence signal in the strain without the repressor (wt + GFP) was temperature-independent and has saturated the fluorimeter detector (260000) after 5 hours of growth. Wt (*E*. *coli* K12 MG1655 wild type strain), wt+GFP (*E*. *coli* K12 MG1655 wild type strain transformed with the plasmid with GFP controlled by pR promoter). flgAi, flgFi, flgGi, flgIi, and flgJi+GFP (*E*. *coli* K12 MG1655 with integrations into *flgA*, *flgF*, *flgG*, *flgI*, and *flgJ* transformed with the plasmid with GFP controlled by pR promoter). All expression experiments were performed in triplicate and the means and standard errors were calculated.

The high probability of the *E*. *coli* K12 MG1655 flagellar region 1 being occupied by RNA polymerase (Fig. 1) suggests that genetic circuits integrated here would be strongly expressed. We verified this by measuring relative expression of *flgA*, *flgF*, *flgG*, *flgI*, and *flgJ* by RT-PCR (Fig. 5A). The relative expression of the two integration target loci, *flgF* and *flgG*, was higher (2–3 fold) than the mean expression of the housekeeping genes *arcA* and *rpoD* [30, 31]. The relative expression of *flgI* and *flgJ* was lower, while the relative expression of *flgA* was not different from the mean expression of the housekeeping genes (Fig. 5A). RT-PCR was also employed to asses the relative expression of the genetic circuit integrated into *flgA*, *flgF*, *flgG*, *flgI*, and *flgJ*. Repr-ts-1 was expressed strongly in all five target loci of the *E*. *coli* flagellar region 1 (Fig. 5B). The highest expression of Repr-ts-1 was detected when integrated into *flgA* and *flgF*. The expression at these sites was 4–5 fold higher than the mean expression of the housekeeping genes (Fig. 5B). Notably, the expression of Repr-ts-1 was significantly higher (3–4 fold) at the best locus of the previously analyzed flagellar region 3a, *fliT* [12] (Fig. 5B). This could be due to the higher probability of the *E*. *coli* K12 MG1655 flagellar region 3a being occupied by RNA polymerase [12–14]. To assess the expression of the integrated genetic circuit we measured the repressive activity of the Repr-ts-1-bourne thermosensitive repressor on the GFP expression (Fig. 6). We introduced plasmid pSB1A1(GFP) into *E*. *coli* harboring chromosomal integrations flgAi, flgFi, flgGi, flgIi, and flgJi. pSB1A1(GFP) harbours GFP-encoding gene downstream of the pR promoter that is controlled by the Repr-ts-1-bourne repressor. The repressive activity of the integrated genetic circuit was quantified by measuring GFP expression over time with the multiwell plate fuorimeter (Fluostar Omega). At 30°C GFP was not expressed, while increased temperature (42°C) led to GFP expression (Fig. 6).

## Conclusions

The *E*. *coli* flagellar genes are suitable targets for integration of genetic circuits. Previous investigation of the *E*. *coli* K12 MG1655 flagellar region 3a led to the identification of one locus that supports both high integration efficiency and strong expression of the integrated genetic circuit [12]. The identification and validation of other suitable loci would significantly aid progress. The aim of this analysis was to identify and validate suitable integration and expression sites within *E*. *coli* flagellar region 1, which has good transcriptional profile and high probability of being occupied by RNA polymerase. We integrated genetic circuit Repr-ts-1 into five open reading frames of the flagellar region 1 (*flgA*, *flgF*, *flgG*, *flgI*, and *flgJ*) that are well-conserved among commonly-used *E*. *coli* strains. The five targeted genes of the flagellar region 1 varied significantly in the efficiency of integration and expression of the integrated genetic circuit. The integrations did not have a negative impact on growth; however, they all abolished motility. This analysis shows that *flgA* and *flgG* are the most suitable chromosomal integration sites of the investigated *E*. *coli* K12 MG1655 flagellar region 1 due to high efficiency of integration and strong relative expression. Expression of the integrated genetic circuit at *flgF* is higher than expression at *flgG*; however, the integration efficiency at *flgF* is only half of that measured at *flgA* and *flgG*. In conclusion, the presented study of the *E*. *coli* flagellar region 1 contributes two novel sites for the robust integration and expression of synthetic gene circuits.

## Supporting Information

S1 Fig Confirmation of the integration into the *E*. *coli* flagellar region 1. Figure depicts chromosomal integration into the target genes of the *E*. *coli* K12 MG1655 flagellar region 1 (*flgA* (flgAi), *flgF* (flgFi), *flgG* (flgGi), *flgI* (flgIi), and *flgJ* (flgJi)). Flanking primers were used for the verification of successful integration and the HyperLadder 1kb (Bioline) has been used as the molecular weight marker. Wt (wild type), +i (integrated DNA fragment).
(TIF)

S1 Table Primers used in this study. (DOC)

S2 Table Raw plate reader data of the growth rates of strains with chromosomal integrations. (DOC)
