# Supplementary figures and images for: Identification and Validation of Novel Chromosomal Integration and Expression Loci in Escherichia coli Flagellar Region 1
Source: PLoS One. 2015 Mar 27;10(3):e0123007. doi: 10.1371/journal.pone.0123007 (PMC4376774; doi:10.1371/journal.pone.0123007)

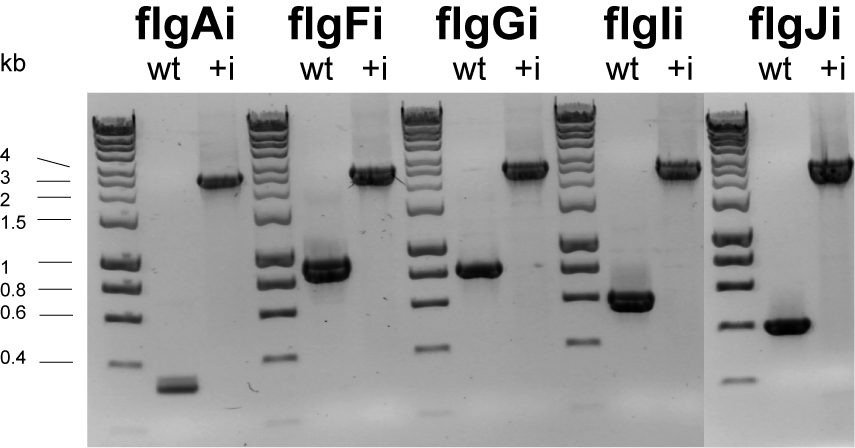

Supplement: S1 Fig — Figure depicts chromosomal integration into the target genes of the E. coli K12 MG1655 flagellar region 1 (flgA (flgAi), flgF (flgFi), flgG (flgGi), flgI (flgIi), and flgJ (flgJi)). Flanking primers were used for the verification of successful integration and the HyperLadder 1kb (Bioline) has been used as the molecular weight marker. Wt (wild type), +i (integrated DNA fragment). (TIF) [file pone.0123007.s001.tif]
