# Supplementary material for: Identification and Validation of Novel Chromosomal Integration and Expression Loci in Escherichia coli Flagellar Region 1
Source: PLoS One. 2015 Mar 27;10(3):e0123007. doi: 10.1371/journal.pone.0123007 (PMC4376774; doi:10.1371/journal.pone.0123007)
Supplement: S1 Table — (DOC) [file pone.0123007.s002.doc]

**S1 Table. Primers used in this study.**

| **Primer (Sequence 5’→ 3’)** |
| --- |
| flgArepF: *flgA* integration primer forward |
| AATGTGAATGTGTTGGCACGCTGCGGTAACGACAAACGATATTTACAGGTTAATGTACAGGCCACAGTATCACGAGGCAGAATTTCAGAT |
| flgArepR: *flgA* integration primer reverse |
| TTAACTGGATAGGTTGATCGGGTGATAGATCGCGCAGGCTAATGGCATCAACAAGTTGATTGATATCGGTTTTAAAGAAAAAGGGCAGG |
| flgFrepF: *flgF* integration primer forward |
| CTCAACACGCTGGTTAACTTACGCTAATCGCTGACGGGATAGCTCAATGGATCACGCAATTTATACGTATCACGAGGCAGAATTTCAGAT |
| flgFrepR: *flgF* integration primer reverse |
| AACTTGCGCATCGGGCAACTTATACTTGTCATGTATCCTTTCAATTAACTCATCGACAGCAGTTGGCGGTTTTAAAGAAAAAGGGCAGG |
| flgGrepF: *flgG* integration primer forward |
| TTTGTCACTAATCCACTACAGGACATTTTATGATCAGTTCATTATGGATCGCCAAAACGGGCCTTGGTATCACGAGGCAGAATTTCAGAT |
| flgGrepR: *flgG* integration primer reverse |
| CACCTGAATCATATTGACCAGTTCTTCCGCCACGTTGACGTTAGACGTTTCAACATACCCTTGATACGGTTTTAAAGAAAAAGGGCAGG |
| flgIrepF: *flgI* integration primer forward |
| TACCCTTAATTTGCAACTTAACGACGAAGATTTCAGCATGGCGCAGCAAATCGCTGACACCATCAAGTATCACGAGGCAGAATTTCAGAT |
| flgIrepR: *flgI* integration primer reverse |
| TCATCAGATGATTTCCAGTTTTGCCCGCAGACATCCCGCACTTTGCATTGATTGCAGTATGGACATCGGTTTTAAAGAAAAAGGGCAGG |
| flgJrepF: *flgJ* integration primer forward |
| TTTGGTGTCAAAGCCTCTGGCAACTGGAAAGGGCCAGTTACTGAAATCACCACGACTGAATATGAAGTATCACGAGGCAGAATTTCAGAT |
| flgJrepR: *flgJ* integration primer reverse |
| ATACTGTAGGTTTTGCTCACCTTGTCGCTTATCGATTTCATCTGCTGAATCATGTTGGTGAGTTTGCGGTTTTAAAGAAAAAGGGCAGG |
| flgArepTF: *flgA* integration test primer forward |
| TGCTTTCGATGAGCAATAATTCCCGCCTG |
| flgArepTR: *flgA* integration test primer reverse |
| GTCCCGCTTTTACCCGCCATGC |
| flgFrepTF: *flgF* integration test primer forward |
| CTAACGCCCAGACCATCAAAACCCAGG |
| flgFrepTR: *flgF* integration test primer reverse |
| CGATAAATTGCGATTGCCCCCATAGACC |
| flgGrepTF: *flgG* integration test primer forward |
| CGCATCCGGCAAGAAGACATATGCAC |
| flgGrepTR: *flgG* integration test primer reverse |
| GCAGCATCTGATCGGTGGTGGACA |
| flgIrepTF: *flgI* integration test primer forward |
| ACGTGAATTGCCCAGCCAGTTTGG |
| flgIrepTR: *flgI* integration test primer reverse |
| CGCACTTGCCAGTAGTTTGCTGTCG |
| flgJrepTF: *flgJ* integration test primer forward |
| GGCAAATCCGCCGCGAAAACG |
| flgJrepTR: *flgJ* integration test primer reverse |
| CCCGCCGGACTTGAGTTATTCAGAACAG |
