# Supplementary material for: Identification and Validation of Novel Chromosomal Integration and Expression Loci in Escherichia coli Flagellar Region 1
Source: PLoS One. 2015 Mar 27;10(3):e0123007. doi: 10.1371/journal.pone.0123007 (PMC4376774; doi:10.1371/journal.pone.0123007)
Supplement: S2 Table — (DOC) [file pone.0123007.s003.doc]

**S2 Table. Raw plate reader data of the growth rates of strains with chromosomal integrations.**

| **wild type 30 °C** | | | | | |
| --- | --- | --- | --- | --- | --- |
| **Time (h)** | **1** | **2** | **3** | **Average** | **StDev** |
| 0 | 0.05 | 0.05 | 0.05 | 0.05 | 0 |
| 0.5 | 0.085 | 0.0787 | 0.0834 | 0.082367 | 0.003275 |
| 1 | 0.0988 | 0.0903 | 0.0954 | 0.094833 | 0.004278 |
| 1.5 | 0.1255 | 0.1151 | 0.12 | 0.1202 | 0.005203 |
| 2 | 0.1728 | 0.1543 | 0.1636 | 0.163567 | 0.00925 |
| 2.5 | 0.2442 | 0.2174 | 0.2269 | 0.2295 | 0.013588 |
| 3 | 0.3331 | 0.2887 | 0.3108 | 0.310867 | 0.0222 |
| 3.5 | 0.459 | 0.4268 | 0.4461 | 0.443967 | 0.016206 |
| 4 | 0.6353 | 0.559 | 0.5899 | 0.594733 | 0.038379 |
| 4.5 | 0.9159 | 0.8223 | 0.8455 | 0.861233 | 0.048743 |
| 5 | 1.076 | 1.0385 | 1.0344 | 1.049633 | 0.022926 |
| 5.5 | 1.2436 | 1.1906 | 1.2017 | 1.211967 | 0.027952 |
| 6 | 1.3511 | 1.2898 | 1.3167 | 1.3192 | 0.030726 |
| 6.5 | 1.4331 | 1.3777 | 1.408 | 1.406267 | 0.027741 |
| 7 | 1.5135 | 1.4764 | 1.4958 | 1.495233 | 0.018556 |
| 7.5 | 1.5859 | 1.5443 | 1.5716 | 1.567267 | 0.021136 |
| 8 | 1.6453 | 1.6171 | 1.6305 | 1.630967 | 0.014106 |
| 8.5 | 1.7695 | 1.7225 | 1.7668 | 1.752933 | 0.026391 |
| 9 | 1.8002 | 1.7811 | 1.79 | 1.790433 | 0.009557 |
| 9.5 | 1.821 | 1.8073 | 1.8142 | 1.814167 | 0.00685 |
| 10 | 1.8358 | 1.8275 | 1.8461 | 1.836467 | 0.009318 |
| 10.5 | 1.8605 | 1.8486 | 1.8686 | 1.859233 | 0.01006 |
| 11 | 1.8733 | 1.8715 | 1.89 | 1.878267 | 0.010201 |
| 11.5 | 1.8829 | 1.8905 | 1.8991 | 1.890833 | 0.008105 |
| 12 | 1.8956 | 1.9149 | 1.9081 | 1.9062 | 0.009789 |
| 12.5 | 1.8934 | 1.9226 | 1.9062 | 1.9074 | 0.014637 |
| 13 | 1.9111 | 1.9386 | 1.9133 | 1.921 | 0.015282 |
| 13.5 | 1.9135 | 1.9452 | 1.9182 | 1.925633 | 0.017107 |
| 14 | 1.9086 | 1.9416 | 1.9116 | 1.9206 | 0.018248 |
| 14.5 | 1.8968 | 1.9505 | 1.9006 | 1.915967 | 0.029967 |
| 15 | 1.9001 | 1.9526 | 1.9001 | 1.9176 | 0.030311 |
| 15.5 | 1.9065 | 1.9629 | 1.901 | 1.923467 | 0.034261 |
| 16 | 1.9044 | 1.9627 | 1.9014 | 1.922833 | 0.034558 |
| 16.5 | 1.9078 | 1.9709 | 1.9012 | 1.926633 | 0.038478 |
| 17 | 1.9785 | 2.0262 | 1.9596 | 1.9881 | 0.034322 |
| 17.5 | 1.8928 | 1.9564 | 1.8884 | 1.912533 | 0.038053 |
| 18 | 1.8957 | 1.9551 | 1.8793 | 1.910033 | 0.039881 |
| 18.5 | 1.8906 | 1.9509 | 1.8783 | 1.9066 | 0.038855 |
| 19 | 1.887 | 1.9479 | 1.8715 | 1.902133 | 0.040386 |
| 19.5 | 1.887 | 1.9484 | 1.8675 | 1.900967 | 0.04222 |
| 20 | 1.8844 | 1.9421 | 1.8661 | 1.897533 | 0.039666 |
| 20.5 | 1.8784 | 1.9339 | 1.8615 | 1.891267 | 0.037876 |
| 21 | 1.874 | 1.9331 | 1.8603 | 1.889133 | 0.038688 |
| 21.5 | 1.8803 | 1.9344 | 1.8589 | 1.8912 | 0.038912 |
| 22 | 1.8747 | 1.9279 | 1.8532 | 1.885267 | 0.038455 |
| 22.5 | 1.8697 | 1.923 | 1.8477 | 1.880133 | 0.038719 |
| 23 | 1.8607 | 1.9151 | 1.8416 | 1.872467 | 0.038137 |
| 23.5 | 1.8599 | 1.9126 | 1.8356 | 1.869367 | 0.039363 |
| 24 | 1.8577 | 1.9097 | 1.832 | 1.866467 | 0.039585 |

| **flgAi 30 °C** | | | | | |
| --- | --- | --- | --- | --- | --- |
| **Time (h)** | **1** | **2** | **3** | **Average** | **StDev** |
| 0 | 0.05 | 0.05 | 0.05 | 0.05 | 0 |
| 0.5 | 0.0837 | 0.0798 | 0.0799 | 0.081442 | 0.001917 |
| 1 | 0.0996 | 0.0919 | 0.0899 | 0.094058 | 0.004214 |
| 1.5 | 0.1145 | 0.1096 | 0.1058 | 0.112525 | 0.006234 |
| 2 | 0.1521 | 0.145 | 0.1432 | 0.150967 | 0.009237 |
| 2.5 | 0.2055 | 0.1985 | 0.1929 | 0.2066 | 0.016113 |
| 3 | 0.2924 | 0.2808 | 0.2726 | 0.289167 | 0.016591 |
| 3.5 | 0.4386 | 0.4185 | 0.404 | 0.426267 | 0.018453 |
| 4 | 0.5759 | 0.5586 | 0.5469 | 0.569033 | 0.020868 |
| 4.5 | 0.8682 | 0.841 | 0.8174 | 0.846958 | 0.022834 |
| 5 | 1.083 | 1.0674 | 1.0707 | 1.067683 | 0.013779 |
| 5.5 | 1.2255 | 1.2141 | 1.2251 | 1.219167 | 0.007137 |
| 6 | 1.3706 | 1.347 | 1.349 | 1.34645 | 0.021076 |
| 6.5 | 1.4785 | 1.4603 | 1.4963 | 1.460342 | 0.038931 |
| 7 | 1.6049 | 1.6066 | 1.6724 | 1.594783 | 0.073431 |
| 7.5 | 1.6838 | 1.7264 | 1.7894 | 1.691717 | 0.093622 |
| 8 | 1.7606 | 1.856 | 1.8742 | 1.780442 | 0.111409 |
| 8.5 | 1.841 | 1.9327 | 1.9572 | 1.870958 | 0.093233 |
| 9 | 1.9084 | 2.0023 | 2.0685 | 1.942408 | 0.120746 |
| 9.5 | 1.8346 | 1.8878 | 2.1691 | 1.926417 | 0.164739 |
| 10 | 1.8837 | 1.9424 | 2.1565 | 1.954767 | 0.141297 |
| 10.5 | 1.9449 | 2.1803 | 2.3149 | 2.074833 | 0.209858 |
| 11 | 1.959 | 2.1206 | 2.4422 | 2.100017 | 0.24938 |
| 11.5 | 2.0059 | 2.2314 | 2.3863 | 2.128608 | 0.222535 |
| 12 | 2.0074 | 2.2029 | 2.4858 | 2.150575 | 0.255168 |
| 12.5 | 2.1708 | 2.3914 | 2.511 | 2.24515 | 0.265624 |
| 13 | 2.1443 | 2.4377 | 2.5514 | 2.2636 | 0.285626 |
| 13.5 | 2.1328 | 2.3972 | 2.5429 | 2.249633 | 0.274716 |
| 14 | 2.2257 | 2.3704 | 2.6502 | 2.291725 | 0.303747 |
| 14.5 | 2.2318 | 2.4073 | 2.6432 | 2.299567 | 0.306285 |
| 15 | 2.203 | 2.3067 | 2.5842 | 2.252875 | 0.275421 |
| 15.5 | 2.2282 | 2.3433 | 2.5858 | 2.270192 | 0.275036 |
| 16 | 2.2099 | 2.343 | 2.587 | 2.265683 | 0.276815 |
| 16.5 | 2.2322 | 2.3217 | 2.5813 | 2.265458 | 0.270079 |
| 17 | 2.1229 | 2.1948 | 2.4973 | 2.200775 | 0.215451 |
| 17.5 | 2.2504 | 2.4906 | 2.4873 | 2.285208 | 0.272718 |
| 18 | 2.1993 | 2.3084 | 2.4518 | 2.217383 | 0.229511 |
| 18.5 | 2.1954 | 2.2051 | 2.2845 | 2.1479 | 0.165744 |
| 19 | 2.1742 | 2.205 | 2.1983 | 2.119908 | 0.145785 |
| 19.5 | 2.1923 | 2.2232 | 2.2006 | 2.129267 | 0.152759 |
| 20 | 2.1842 | 2.2175 | 2.2032 | 2.125608 | 0.152661 |
| 20.5 | 2.168 | 2.19 | 2.1941 | 2.110842 | 0.146831 |
| 21 | 2.17 | 2.1873 | 2.1854 | 2.107958 | 0.146089 |
| 21.5 | 2.1331 | 2.167 | 2.1732 | 2.091125 | 0.134444 |
| 22 | 2.1304 | 2.1564 | 2.1436 | 2.078917 | 0.129536 |
| 22.5 | 2.1277 | 2.1519 | 2.1348 | 2.073633 | 0.129399 |
| 23 | 2.1134 | 2.1409 | 2.127 | 2.063442 | 0.127811 |
| 23.5 | 2.0997 | 2.1259 | 2.1053 | 2.050067 | 0.120992 |
| 24 | 2.0855 | 2.1223 | 2.0787 | 2.038242 | 0.116107 |

| **flgFi 30 °C** | | | | | |
| --- | --- | --- | --- | --- | --- |
| **Time (h)** | **1** | **2** | **3** | **Average** | **StDev** |
| 0 | 0.05 | 0.05 | 0.05 | 0.05 | 0 |
| 0.5 | 0.0823 | 0.0913 | 0.0795 | 0.083867 | 0.005132 |
| 1 | 0.0929 | 0.0935 | 0.0927 | 0.093483 | 0.000962 |
| 1.5 | 0.1174 | 0.1184 | 0.1146 | 0.11765 | 0.00234 |
| 2 | 0.1657 | 0.162 | 0.1578 | 0.162267 | 0.003342 |
| 2.5 | 0.2291 | 0.2269 | 0.2177 | 0.2258 | 0.00552 |
| 3 | 0.3274 | 0.3205 | 0.307 | 0.316442 | 0.009252 |
| 3.5 | 0.4869 | 0.4827 | 0.4505 | 0.466017 | 0.02192 |
| 4 | 0.6377 | 0.6328 | 0.6067 | 0.617983 | 0.020625 |
| 4.5 | 0.9551 | 0.9377 | 0.8889 | 0.910733 | 0.043292 |
| 5 | 1.1502 | 1.1488 | 1.124 | 1.118158 | 0.047242 |
| 5.5 | 1.2977 | 1.2749 | 1.2783 | 1.265717 | 0.037214 |
| 6 | 1.4867 | 1.4503 | 1.4666 | 1.4307 | 0.075809 |
| 6.5 | 1.7056 | 1.6173 | 1.6686 | 1.599442 | 0.133776 |
| 7 | 1.8444 | 1.7673 | 1.8003 | 1.726808 | 0.157581 |
| 7.5 | 1.9256 | 1.8795 | 1.8959 | 1.817067 | 0.167623 |
| 8 | 2.022 | 1.9635 | 1.9687 | 1.896292 | 0.178848 |
| 8.5 | 2.2186 | 2.0672 | 2.1157 | 2.038608 | 0.200639 |
| 9 | 2.1434 | 2.1609 | 2.3133 | 2.102008 | 0.221288 |
| 9.5 | 2.3809 | 2.1916 | 2.3402 | 2.181717 | 0.258187 |
| 10 | 2.301 | 2.311 | 2.3615 | 2.202492 | 0.245449 |
| 10.5 | 2.5179 | 2.415 | 2.5458 | 2.334483 | 0.321788 |
| 11 | 2.5059 | 2.4885 | 2.4833 | 2.338992 | 0.307302 |
| 11.5 | 2.4866 | 2.5416 | 2.5822 | 2.375308 | 0.325351 |
| 12 | 2.3594 | 2.4219 | 2.5803 | 2.31695 | 0.289186 |
| 12.5 | 2.5071 | 2.5804 | 2.5872 | 2.395525 | 0.327431 |
| 13 | 2.4523 | 2.5889 | 2.5852 | 2.38685 | 0.317 |
| 13.5 | 2.4018 | 2.4346 | 2.4364 | 2.299608 | 0.249823 |
| 14 | 2.5691 | 2.6674 | 2.6111 | 2.44205 | 0.349958 |
| 14.5 | 2.5367 | 2.5376 | 2.5368 | 2.381767 | 0.310534 |
| 15 | 2.4801 | 2.5151 | 2.618 | 2.3827 | 0.315542 |
| 15.5 | 2.4951 | 2.444 | 2.5611 | 2.355917 | 0.292258 |
| 16 | 2.372 | 2.6404 | 2.5533 | 2.372133 | 0.319718 |
| 16.5 | 2.4054 | 2.6194 | 2.5763 | 2.381933 | 0.317289 |
| 17 | 2.4804 | 2.63 | 2.548 | 2.411625 | 0.2889 |
| 17.5 | 2.5027 | 2.3282 | 2.7036 | 2.361758 | 0.336477 |
| 18 | 2.3081 | 2.5735 | 2.4831 | 2.318683 | 0.293866 |
| 18.5 | 2.4396 | 2.5712 | 2.5014 | 2.3547 | 0.303532 |
| 19 | 2.2898 | 2.5828 | 2.4661 | 2.310208 | 0.297518 |
| 19.5 | 2.6148 | 2.6293 | 2.5994 | 2.436117 | 0.356975 |
| 20 | 2.3935 | 2.5909 | 2.461 | 2.335733 | 0.303402 |
| 20.5 | 2.5121 | 2.5494 | 2.6597 | 2.403117 | 0.34694 |
| 21 | 2.5048 | 2.5626 | 2.4514 | 2.351983 | 0.31189 |
| 21.5 | 2.5086 | 2.6003 | 2.4071 | 2.3518 | 0.317043 |
| 22 | 2.5114 | 2.6505 | 2.5613 | 2.402117 | 0.349338 |
| 22.5 | 2.5733 | 2.7009 | 2.3776 | 2.382983 | 0.360638 |
| 23 | 2.4218 | 2.4963 | 2.5856 | 2.344042 | 0.321436 |
| 23.5 | 2.5548 | 2.4878 | 2.5593 | 2.367817 | 0.333905 |
| 24 | 2.5754 | 2.5899 | 2.4587 | 2.372617 | 0.342506 |

| **flgGi 30 °C** | | | | | |
| --- | --- | --- | --- | --- | --- |
| **Time (h)** | **1** | **2** | **3** | **Average** | **StDev** |
| 0 | 0.05 | 0.05 | 0.05 | 0.05 | 0 |
| 0.5 | 0.0734 | 0.0776 | 0.0805 | 0.078467 | 0.003906 |
| 1 | 0.0835 | 0.0873 | 0.0877 | 0.088333 | 0.004729 |
| 1.5 | 0.1048 | 0.1117 | 0.1117 | 0.1121 | 0.006304 |
| 2 | 0.1415 | 0.1542 | 0.1541 | 0.153342 | 0.009057 |
| 2.5 | 0.1975 | 0.2139 | 0.2135 | 0.2136 | 0.013065 |
| 3 | 0.2774 | 0.3036 | 0.3048 | 0.299167 | 0.014856 |
| 3.5 | 0.411 | 0.4563 | 0.4563 | 0.441892 | 0.021399 |
| 4 | 0.5631 | 0.605 | 0.6072 | 0.592508 | 0.020344 |
| 4.5 | 0.8532 | 0.9111 | 0.9129 | 0.884608 | 0.031807 |
| 5 | 1.0831 | 1.1211 | 1.1236 | 1.094358 | 0.035106 |
| 5.5 | 1.2232 | 1.2522 | 1.2572 | 1.236142 | 0.022009 |
| 6 | 1.3556 | 1.3933 | 1.4105 | 1.36965 | 0.040705 |
| 6.5 | 1.4841 | 1.5251 | 1.5429 | 1.489592 | 0.060761 |
| 7 | 1.6178 | 1.6422 | 1.6899 | 1.611283 | 0.082959 |
| 7.5 | 1.6843 | 1.7066 | 1.7752 | 1.683342 | 0.086512 |
| 8 | 1.7177 | 1.7589 | 1.8868 | 1.748592 | 0.106455 |
| 8.5 | 1.7571 | 1.8057 | 1.9891 | 1.826208 | 0.111205 |
| 9 | 1.7856 | 1.8051 | 1.8622 | 1.810833 | 0.035234 |
| 9.5 | 1.7939 | 1.7895 | 1.9173 | 1.828717 | 0.060025 |
| 10 | 1.9037 | 1.8289 | 2.0452 | 1.903567 | 0.100229 |
| 10.5 | 2.0599 | 2.0135 | 2.2223 | 2.038733 | 0.149445 |
| 11 | 2.1175 | 1.9469 | 2.186 | 2.032167 | 0.143645 |
| 11.5 | 2.2192 | 2.1129 | 2.2703 | 2.123308 | 0.16828 |
| 12 | 2.2682 | 2.0498 | 2.2738 | 2.1245 | 0.179049 |
| 12.5 | 2.4341 | 2.1825 | 2.4455 | 2.242375 | 0.254173 |
| 13 | 2.3795 | 2.2424 | 2.3586 | 2.225375 | 0.21169 |
| 13.5 | 2.3286 | 2.1129 | 2.1849 | 2.138008 | 0.167588 |
| 14 | 2.4443 | 2.3601 | 2.4711 | 2.299025 | 0.256678 |
| 14.5 | 2.4479 | 2.2788 | 2.3891 | 2.257942 | 0.238515 |
| 15 | 2.3966 | 2.2314 | 2.2949 | 2.210125 | 0.206546 |
| 15.5 | 2.3882 | 2.3171 | 2.4416 | 2.267592 | 0.235017 |
| 16 | 2.3965 | 2.3119 | 2.3011 | 2.233083 | 0.211186 |
| 16.5 | 2.4333 | 2.3408 | 2.4064 | 2.276783 | 0.236644 |
| 17 | 2.4167 | 2.383 | 2.3852 | 2.29325 | 0.204015 |
| 17.5 | 2.3934 | 2.325 | 2.3872 | 2.254533 | 0.230083 |
| 18 | 2.4297 | 2.3906 | 2.3789 | 2.277308 | 0.245812 |
| 18.5 | 2.4006 | 2.1731 | 2.2675 | 2.18695 | 0.208904 |
| 19 | 2.3257 | 2.2904 | 2.5467 | 2.266233 | 0.267925 |
| 19.5 | 2.4315 | 2.3736 | 2.5215 | 2.306892 | 0.277374 |
| 20 | 2.4225 | 2.3422 | 2.5052 | 2.291858 | 0.271175 |
| 20.5 | 2.4368 | 2.4349 | 2.4857 | 2.312167 | 0.281583 |
| 21 | 2.3793 | 2.4964 | 2.395 | 2.289958 | 0.27221 |
| 21.5 | 2.3433 | 2.3522 | 2.4844 | 2.267775 | 0.259208 |
| 22 | 2.3671 | 2.5105 | 2.5457 | 2.327142 | 0.304542 |
| 22.5 | 2.3571 | 2.3974 | 2.4778 | 2.278108 | 0.270019 |
| 23 | 2.3028 | 2.4047 | 2.4894 | 2.267342 | 0.274081 |
| 23.5 | 2.2693 | 2.4898 | 2.5015 | 2.282492 | 0.295402 |
| 24 | 2.2334 | 2.4256 | 2.4814 | 2.251717 | 0.277935 |

| **flgIi 30 °C** | | | | | |
| --- | --- | --- | --- | --- | --- |
| **Time (h)** | **1** | **2** | **3** | **Average** | **StDev** |
| 0 | 0.05 | 0.05 | 0.05 | 0.05 | 0 |
| 0.5 | 0.0913 | 0.0804 | 0.0816 | 0.083917 | 0.004988 |
| 1 | 0.0862 | 0.0921 | 0.0886 | 0.090433 | 0.003804 |
| 1.5 | 0.1077 | 0.1153 | 0.1116 | 0.1137 | 0.00533 |
| 2 | 0.1459 | 0.1593 | 0.1516 | 0.155092 | 0.007879 |
| 2.5 | 0.2013 | 0.2214 | 0.2061 | 0.214575 | 0.013132 |
| 3 | 0.2879 | 0.3147 | 0.2904 | 0.300967 | 0.013772 |
| 3.5 | 0.4137 | 0.4672 | 0.431 | 0.438967 | 0.022539 |
| 4 | 0.5614 | 0.619 | 0.5798 | 0.588733 | 0.024352 |
| 4.5 | 0.8355 | 0.9114 | 0.8608 | 0.867233 | 0.031807 |
| 5 | 1.0757 | 1.1242 | 1.093 | 1.085633 | 0.031285 |
| 5.5 | 1.2117 | 1.2569 | 1.228 | 1.227142 | 0.021253 |
| 6 | 1.3422 | 1.3945 | 1.3751 | 1.35775 | 0.033563 |
| 6.5 | 1.4708 | 1.5268 | 1.5224 | 1.481567 | 0.056271 |
| 7 | 1.6071 | 1.639 | 1.6442 | 1.596383 | 0.069399 |
| 7.5 | 1.6789 | 1.7001 | 1.7318 | 1.669517 | 0.071549 |
| 8 | 1.7214 | 1.7523 | 1.8481 | 1.738192 | 0.089551 |
| 8.5 | 1.7681 | 1.789 | 1.9232 | 1.808308 | 0.078009 |
| 9 | 1.7842 | 1.7971 | 1.8126 | 1.796083 | 0.012206 |
| 9.5 | 1.8145 | 1.7964 | 1.812 | 1.809267 | 0.008649 |
| 10 | 1.862 | 1.8067 | 1.904 | 1.852292 | 0.041219 |
| 10.5 | 1.887 | 1.841 | 2.0673 | 1.913633 | 0.104176 |
| 11 | 1.8972 | 1.8742 | 2.022 | 1.917917 | 0.070109 |
| 11.5 | 1.9028 | 1.9052 | 2.141 | 1.959958 | 0.120858 |
| 12 | 1.958 | 1.9054 | 2.1307 | 1.975075 | 0.106629 |
| 12.5 | 2.0556 | 1.9927 | 2.3314 | 2.071775 | 0.183429 |
| 13 | 2.0965 | 1.9878 | 2.295 | 2.075075 | 0.163485 |
| 13.5 | 2.0593 | 1.9834 | 2.1787 | 2.036758 | 0.109318 |
| 14 | 2.1371 | 2.0814 | 2.3811 | 2.13005 | 0.190886 |
| 14.5 | 2.1507 | 2.024 | 2.26 | 2.087667 | 0.149673 |
| 15 | 2.1735 | 2.0529 | 2.2869 | 2.107725 | 0.158728 |
| 15.5 | 2.2805 | 2.1299 | 2.3225 | 2.164092 | 0.180475 |
| 16 | 2.2841 | 2.0863 | 2.2452 | 2.134608 | 0.165086 |
| 16.5 | 2.298 | 2.0877 | 2.2611 | 2.143358 | 0.171119 |
| 17 | 2.3184 | 2.1112 | 2.3175 | 2.1838 | 0.162852 |
| 17.5 | 2.3937 | 2.121 | 2.2792 | 2.176608 | 0.208552 |
| 18 | 2.4723 | 2.1291 | 2.3824 | 2.223458 | 0.254508 |
| 18.5 | 2.3749 | 2.1014 | 2.3256 | 2.177125 | 0.216085 |
| 19 | 2.4221 | 2.1466 | 2.353 | 2.205958 | 0.233931 |
| 19.5 | 2.3142 | 2.1676 | 2.4386 | 2.205342 | 0.231177 |
| 20 | 2.4226 | 2.1622 | 2.4678 | 2.237533 | 0.263659 |
| 20.5 | 2.3924 | 2.2094 | 2.4501 | 2.235792 | 0.251561 |
| 21 | 2.4767 | 2.2167 | 2.4275 | 2.252508 | 0.267213 |
| 21.5 | 2.3371 | 2.1787 | 2.3345 | 2.185375 | 0.209636 |
| 22 | 2.3024 | 2.2424 | 2.5021 | 2.233042 | 0.257059 |
| 22.5 | 2.3032 | 2.3085 | 2.4132 | 2.226258 | 0.236244 |
| 23 | 2.3333 | 2.2301 | 2.4224 | 2.214567 | 0.241223 |
| 23.5 | 2.3166 | 2.2212 | 2.4094 | 2.204142 | 0.236039 |
| 24 | 2.2395 | 2.2368 | 2.419 | 2.190442 | 0.232203 |

| **flgJi 30 °C** | | | | | |
| --- | --- | --- | --- | --- | --- |
| **Time (h)** | **1** | **2** | **3** | **Average** | **StDev** |
| 0 | 0.05 | 0.05 | 0.05 | 0.05 | 0 |
| 0.5 | 0.0798 | 0.0824 | 0.0879 | 0.083117 | 0.003414 |
| 1 | 0.0889 | 0.0902 | 0.094 | 0.091983 | 0.00288 |
| 1.5 | 0.1117 | 0.1147 | 0.1192 | 0.11645 | 0.003969 |
| 2 | 0.1554 | 0.1598 | 0.1633 | 0.160517 | 0.003819 |
| 2.5 | 0.2187 | 0.2247 | 0.2276 | 0.225125 | 0.004716 |
| 3 | 0.3144 | 0.3226 | 0.3201 | 0.316992 | 0.005334 |
| 3.5 | 0.4607 | 0.4718 | 0.4732 | 0.462417 | 0.013511 |
| 4 | 0.6051 | 0.6272 | 0.6178 | 0.611208 | 0.014235 |
| 4.5 | 0.8883 | 0.9168 | 0.8822 | 0.887133 | 0.022925 |
| 5 | 1.0804 | 1.0976 | 1.0964 | 1.081008 | 0.022338 |
| 5.5 | 1.2155 | 1.2315 | 1.2308 | 1.222442 | 0.010162 |
| 6 | 1.345 | 1.3615 | 1.3549 | 1.34515 | 0.018581 |
| 6.5 | 1.4809 | 1.4931 | 1.4862 | 1.466617 | 0.040542 |
| 7 | 1.6059 | 1.6182 | 1.6049 | 1.581058 | 0.057535 |
| 7.5 | 1.6875 | 1.6951 | 1.6841 | 1.658492 | 0.06099 |
| 8 | 1.7277 | 1.727 | 1.7245 | 1.702542 | 0.047736 |
| 8.5 | 1.7782 | 1.7843 | 1.7873 | 1.775683 | 0.015632 |
| 9 | 1.795 | 1.791 | 1.7954 | 1.792958 | 0.002604 |
| 9.5 | 1.8028 | 1.7992 | 1.8026 | 1.804692 | 0.006529 |
| 10 | 1.8196 | 1.8145 | 1.8058 | 1.819092 | 0.012909 |
| 10.5 | 1.8285 | 1.828 | 1.8101 | 1.831458 | 0.020399 |
| 11 | 1.8343 | 1.8241 | 1.8147 | 1.837842 | 0.028113 |
| 11.5 | 1.845 | 1.8317 | 1.8254 | 1.848233 | 0.029552 |
| 12 | 1.8512 | 1.8297 | 1.8324 | 1.854875 | 0.035528 |
| 12.5 | 1.847 | 1.825 | 1.8333 | 1.853175 | 0.037271 |
| 13 | 1.8543 | 1.8247 | 1.8478 | 1.86195 | 0.041365 |
| 13.5 | 1.8423 | 1.8172 | 1.8485 | 1.858408 | 0.046815 |
| 14 | 1.8421 | 1.8141 | 1.8395 | 1.854075 | 0.046114 |
| 14.5 | 1.8294 | 1.8066 | 1.8261 | 1.844517 | 0.048684 |
| 15 | 1.818 | 1.7878 | 1.8245 | 1.836975 | 0.056078 |
| 15.5 | 1.815 | 1.7941 | 1.8257 | 1.839567 | 0.057452 |
| 16 | 1.8064 | 1.7834 | 1.8242 | 1.834208 | 0.061399 |
| 16.5 | 1.8064 | 1.7825 | 1.8161 | 1.832908 | 0.064059 |
| 17 | 1.807 | 1.7834 | 1.8216 | 1.850025 | 0.093386 |
| 17.5 | 1.7824 | 1.7603 | 1.7983 | 1.813383 | 0.067912 |
| 18 | 1.7803 | 1.7565 | 1.7989 | 1.811433 | 0.067985 |
| 18.5 | 1.7705 | 1.7523 | 1.7963 | 1.806425 | 0.06918 |
| 19 | 1.763 | 1.7462 | 1.7858 | 1.799283 | 0.070461 |
| 19.5 | 1.7718 | 1.7502 | 1.7831 | 1.801517 | 0.06769 |
| 20 | 1.7576 | 1.7393 | 1.7804 | 1.793708 | 0.071229 |
| 20.5 | 1.7546 | 1.7326 | 1.7788 | 1.789317 | 0.070537 |
| 21 | 1.7416 | 1.7215 | 1.7656 | 1.779458 | 0.075306 |
| 21.5 | 1.7358 | 1.7132 | 1.7667 | 1.776725 | 0.079405 |
| 22 | 1.7382 | 1.7177 | 1.7588 | 1.774992 | 0.075407 |
| 22.5 | 1.732 | 1.712 | 1.7471 | 1.767808 | 0.076251 |
| 23 | 1.7205 | 1.6981 | 1.739 | 1.757517 | 0.078437 |
| 23.5 | 1.7198 | 1.6999 | 1.7373 | 1.756592 | 0.07672 |
| 24 | 1.7046 | 1.6888 | 1.7353 | 1.748792 | 0.080791 |

| **wild type 37 °C** | | | | | |
| --- | --- | --- | --- | --- | --- |
| **Time (h)** | **1** | **2** | **3** | **Average** | **StDev** |
| 0 | 0.05 | 0.05 | 0.05 | 0.05 | 0 |
| 0.5 | 0.0897 | 0.09 | 0.0918 | 0.0905 | 0.001135782 |
| 1 | 0.107 | 0.1019 | 0.1087 | 0.105867 | 0.003538832 |
| 1.5 | 0.1576 | 0.1445 | 0.1524 | 0.1515 | 0.006596211 |
| 2 | 0.2568 | 0.2375 | 0.2476 | 0.2473 | 0.009653497 |
| 2.5 | 0.3995 | 0.3964 | 0.4102 | 0.402033 | 0.007240396 |
| 3 | 0.6323 | 0.5998 | 0.6058 | 0.612633 | 0.017294026 |
| 3.5 | 0.9019 | 0.8876 | 0.9106 | 0.900033 | 0.011613067 |
| 4 | 1.0558 | 1.0381 | 1.095 | 1.062967 | 0.029119123 |
| 4.5 | 1.1758 | 1.1601 | 1.2166 | 1.184167 | 0.029164419 |
| 5 | 1.2878 | 1.2759 | 1.3365 | 1.300067 | 0.032108306 |
| 5.5 | 1.3744 | 1.3626 | 1.4178 | 1.384933 | 0.029068425 |
| 6 | 1.4421 | 1.4345 | 1.4858 | 1.454133 | 0.027686158 |
| 6.5 | 1.5055 | 1.5005 | 1.5442 | 1.516733 | 0.023917845 |
| 7 | 1.5956 | 1.5606 | 1.6125 | 1.589567 | 0.026470802 |
| 7.5 | 1.7412 | 1.7193 | 1.7522 | 1.737567 | 0.016748234 |
| 8 | 1.8075 | 1.8038 | 1.8186 | 1.809967 | 0.007702164 |
| 8.5 | 1.8318 | 1.8543 | 1.8448 | 1.843633 | 0.011295279 |
| 9 | 1.8434 | 1.8694 | 1.8442 | 1.852333 | 0.014785579 |
| 9.5 | 1.848 | 1.876 | 1.8412 | 1.855067 | 0.018444873 |
| 10 | 1.8557 | 1.8778 | 1.8359 | 1.856467 | 0.020960518 |
| 10.5 | 1.8621 | 1.8764 | 1.8321 | 1.856867 | 0.022608922 |
| 11 | 1.8654 | 1.8656 | 1.8268 | 1.8526 | 0.022343679 |
| 11.5 | 1.872 | 1.864 | 1.8251 | 1.8537 | 0.025089241 |
| 12 | 1.8714 | 1.8502 | 1.8142 | 1.845267 | 0.028917353 |
| 12.5 | 1.867 | 1.8424 | 1.8042 | 1.837867 | 0.031644483 |
| 13 | 1.8692 | 1.8462 | 1.8078 | 1.841067 | 0.031020208 |
| 13.5 | 1.8581 | 1.838 | 1.7985 | 1.831533 | 0.030321664 |
| 14 | 1.861 | 1.8305 | 1.7958 | 1.8291 | 0.032622538 |
| 14.5 | 1.8517 | 1.8244 | 1.782 | 1.819367 | 0.035121551 |
| 15 | 1.8497 | 1.8197 | 1.7779 | 1.815767 | 0.036061244 |
| 15.5 | 1.8366 | 1.8194 | 1.7793 | 1.811767 | 0.029402778 |
| 16 | 1.8332 | 1.8147 | 1.7701 | 1.806 | 0.03243717 |
| 16.5 | 1.8272 | 1.8043 | 1.7553 | 1.7956 | 0.03673105 |
| 17 | 1.8277 | 1.7939 | 1.7539 | 1.791833 | 0.03694338 |
| 17.5 | 1.832 | 1.7893 | 1.7492 | 1.790167 | 0.041406803 |
| 18 | 1.8147 | 1.7789 | 1.744 | 1.7792 | 0.035350955 |
| 18.5 | 1.814 | 1.7754 | 1.7386 | 1.776 | 0.037703581 |
| 19 | 1.8044 | 1.7657 | 1.7292 | 1.766433 | 0.037605363 |
| 19.5 | 1.8061 | 1.771 | 1.7299 | 1.769 | 0.03813935 |
| 20 | 1.7994 | 1.7651 | 1.7286 | 1.764367 | 0.035405696 |
| 20.5 | 1.7959 | 1.7547 | 1.7198 | 1.7568 | 0.038093438 |
| 21 | 1.7894 | 1.7488 | 1.7182 | 1.752133 | 0.035716849 |
| 21.5 | 1.7832 | 1.7443 | 1.7092 | 1.745567 | 0.037016258 |
| 22 | 1.7793 | 1.7401 | 1.7065 | 1.741967 | 0.03643588 |
| 22.5 | 1.7773 | 1.7369 | 1.7057 | 1.739967 | 0.035898375 |
| 23 | 1.7719 | 1.7327 | 1.7022 | 1.7356 | 0.034940378 |
| 23.5 | 1.764 | 1.7221 | 1.6918 | 1.725967 | 0.036254977 |
| 24 | 1.762 | 1.7119 | 1.6888 | 1.7209 | 0.037420716 |

| **flgAi 37 °C** | | | | | |
| --- | --- | --- | --- | --- | --- |
| **Time (h)** | **1** | **2** | **3** | **Average** | **StDev** |
| 0 | 0.05 | 0.05 | 0.05 | 0.05 | 0 |
| 0.5 | 0.1057 | 0.0848 | 0.0825 | 0.090875 | 0.01044 |
| 1 | 0.1058 | 0.1027 | 0.0971 | 0.102867 | 0.004119 |
| 1.5 | 0.1565 | 0.1527 | 0.1411 | 0.15045 | 0.006588 |
| 2 | 0.2522 | 0.2536 | 0.2268 | 0.244975 | 0.012414 |
| 2.5 | 0.4114 | 0.4125 | 0.3748 | 0.400183 | 0.017562 |
| 3 | 0.6302 | 0.6326 | 0.5653 | 0.610183 | 0.031218 |
| 3.5 | 0.8978 | 0.9147 | 0.8507 | 0.890808 | 0.02777 |
| 4 | 1.0904 | 1.0998 | 1.079 | 1.083042 | 0.015857 |
| 4.5 | 1.234 | 1.2508 | 1.218 | 1.221742 | 0.028405 |
| 5 | 1.3813 | 1.3961 | 1.3705 | 1.361992 | 0.042596 |
| 5.5 | 1.4906 | 1.5026 | 1.4884 | 1.466633 | 0.054823 |
| 6 | 1.5766 | 1.5883 | 1.5772 | 1.549058 | 0.063512 |
| 6.5 | 1.652 | 1.6589 | 1.6494 | 1.619258 | 0.068467 |
| 7 | 1.7095 | 1.7163 | 1.7082 | 1.680892 | 0.060987 |
| 7.5 | 1.7744 | 1.7776 | 1.7753 | 1.766217 | 0.019147 |
| 8 | 1.8063 | 1.8121 | 1.8126 | 1.810242 | 0.002865 |
| 8.5 | 1.8253 | 1.8287 | 1.8355 | 1.833283 | 0.008099 |
| 9 | 1.8201 | 1.8275 | 1.8323 | 1.833058 | 0.013795 |
| 9.5 | 1.8195 | 1.8212 | 1.832 | 1.831942 | 0.01638 |
| 10 | 1.8216 | 1.8254 | 1.837 | 1.835117 | 0.015668 |
| 10.5 | 1.8217 | 1.8283 | 1.8361 | 1.835742 | 0.015264 |
| 11 | 1.8147 | 1.8188 | 1.8232 | 1.827325 | 0.017204 |
| 11.5 | 1.8049 | 1.8145 | 1.8174 | 1.822625 | 0.021394 |
| 12 | 1.8032 | 1.8041 | 1.8059 | 1.814617 | 0.020464 |
| 12.5 | 1.803 | 1.8021 | 1.8089 | 1.812967 | 0.016872 |
| 13 | 1.811 | 1.8006 | 1.8066 | 1.814817 | 0.018012 |
| 13.5 | 1.8096 | 1.7884 | 1.7962 | 1.806433 | 0.018885 |
| 14 | 1.797 | 1.7826 | 1.7911 | 1.79995 | 0.020312 |
| 14.5 | 1.7965 | 1.7744 | 1.7749 | 1.791292 | 0.021365 |
| 15 | 1.788 | 1.7724 | 1.7735 | 1.787417 | 0.020193 |
| 15.5 | 1.7788 | 1.7621 | 1.7688 | 1.780367 | 0.022029 |
| 16 | 1.7786 | 1.7532 | 1.7634 | 1.7753 | 0.022974 |
| 16.5 | 1.7814 | 1.7587 | 1.749 | 1.771175 | 0.021201 |
| 17 | 1.7604 | 1.7332 | 1.746 | 1.757858 | 0.025228 |
| 17.5 | 1.7578 | 1.7335 | 1.7378 | 1.754817 | 0.025836 |
| 18 | 1.7474 | 1.7182 | 1.7353 | 1.745025 | 0.025741 |
| 18.5 | 1.7384 | 1.7147 | 1.7311 | 1.74005 | 0.025935 |
| 19 | 1.7393 | 1.7166 | 1.72 | 1.735583 | 0.022867 |
| 19.5 | 1.7311 | 1.7085 | 1.7158 | 1.7311 | 0.026965 |
| 20 | 1.7253 | 1.6973 | 1.7105 | 1.724367 | 0.029016 |
| 20.5 | 1.7205 | 1.6889 | 1.7028 | 1.71725 | 0.029367 |
| 21 | 1.7142 | 1.6857 | 1.7018 | 1.713458 | 0.0283 |
| 21.5 | 1.7059 | 1.6783 | 1.6933 | 1.705767 | 0.028832 |
| 22 | 1.7067 | 1.6719 | 1.6869 | 1.701867 | 0.030295 |
| 22.5 | 1.6973 | 1.6663 | 1.6827 | 1.696567 | 0.031583 |
| 23 | 1.6982 | 1.6632 | 1.6813 | 1.694575 | 0.030859 |
| 23.5 | 1.6869 | 1.652 | 1.6706 | 1.683867 | 0.031481 |
| 24 | 1.6853 | 1.6548 | 1.6535 | 1.678625 | 0.031784 |

| **flgFi 37 °C** | | | | | |
| --- | --- | --- | --- | --- | --- |
| **Time (h)** | **1** | **2** | **3** | **Average** | **StDev** |
| 0 | 0.05 | 0.05 | 0.05 | 0.05 | 0 |
| 0.5 | 0.0815 | 0.0892 | 0.0879 | 0.087275 | 0.003994 |
| 1 | 0.0978 | 0.1055 | 0.1026 | 0.102942 | 0.003726 |
| 1.5 | 0.1441 | 0.1603 | 0.1592 | 0.153775 | 0.007545 |
| 2 | 0.2408 | 0.2665 | 0.2719 | 0.256625 | 0.014925 |
| 2.5 | 0.4081 | 0.4342 | 0.4449 | 0.422308 | 0.020532 |
| 3 | 0.6388 | 0.6685 | 0.6894 | 0.652333 | 0.033638 |
| 3.5 | 0.917 | 0.9502 | 0.9653 | 0.933133 | 0.029899 |
| 4 | 1.099 | 1.1361 | 1.1545 | 1.113142 | 0.040641 |
| 4.5 | 1.2514 | 1.2891 | 1.3185 | 1.260792 | 0.057998 |
| 5 | 1.4045 | 1.4448 | 1.4674 | 1.404192 | 0.074132 |
| 5.5 | 1.5099 | 1.5485 | 1.5627 | 1.501508 | 0.080855 |
| 6 | 1.5999 | 1.6312 | 1.6471 | 1.583083 | 0.088175 |
| 6.5 | 1.6829 | 1.7183 | 1.7305 | 1.662108 | 0.098997 |
| 7 | 1.7672 | 1.8006 | 1.8096 | 1.741742 | 0.103077 |
| 7.5 | 1.8067 | 1.8573 | 1.8621 | 1.815917 | 0.057934 |
| 8 | 1.828 | 1.8753 | 1.8745 | 1.846942 | 0.033114 |
| 8.5 | 1.8417 | 1.8911 | 1.8924 | 1.867208 | 0.028354 |
| 9 | 1.8381 | 1.8806 | 1.8832 | 1.863558 | 0.021987 |
| 9.5 | 1.8311 | 1.8763 | 1.8784 | 1.860217 | 0.022088 |
| 10 | 1.8338 | 1.8749 | 1.8805 | 1.861417 | 0.02108 |
| 10.5 | 1.8336 | 1.8746 | 1.8784 | 1.860867 | 0.020457 |
| 11 | 1.8177 | 1.8583 | 1.8676 | 1.84905 | 0.021795 |
| 11.5 | 1.8125 | 1.8446 | 1.853 | 1.84095 | 0.019412 |
| 12 | 1.8136 | 1.8394 | 1.8523 | 1.837642 | 0.016873 |
| 12.5 | 1.8091 | 1.8408 | 1.8491 | 1.834217 | 0.017407 |
| 13 | 1.8092 | 1.8332 | 1.8442 | 1.831917 | 0.015836 |
| 13.5 | 1.801 | 1.8184 | 1.8279 | 1.819708 | 0.013646 |
| 14 | 1.7908 | 1.8004 | 1.8187 | 1.80975 | 0.017331 |
| 14.5 | 1.7918 | 1.7984 | 1.819 | 1.807142 | 0.014164 |
| 15 | 1.7865 | 1.784 | 1.8078 | 1.798517 | 0.015694 |
| 15.5 | 1.7783 | 1.7709 | 1.8005 | 1.790367 | 0.019019 |
| 16 | 1.7727 | 1.7565 | 1.7886 | 1.78095 | 0.021228 |
| 16.5 | 1.7788 | 1.7498 | 1.7883 | 1.778125 | 0.020097 |
| 17 | 1.76 | 1.7291 | 1.7719 | 1.763208 | 0.026259 |
| 17.5 | 1.7584 | 1.7234 | 1.7692 | 1.760292 | 0.027907 |
| 18 | 1.7483 | 1.7163 | 1.7589 | 1.750675 | 0.026259 |
| 18.5 | 1.7424 | 1.7035 | 1.7493 | 1.7428 | 0.02994 |
| 19 | 1.7399 | 1.6939 | 1.7484 | 1.737158 | 0.030888 |
| 19.5 | 1.7373 | 1.6892 | 1.7326 | 1.732025 | 0.032809 |
| 20 | 1.728 | 1.675 | 1.7233 | 1.722667 | 0.036696 |
| 20.5 | 1.7263 | 1.6689 | 1.7173 | 1.717325 | 0.036441 |
| 21 | 1.7212 | 1.6646 | 1.7133 | 1.712808 | 0.036245 |
| 21.5 | 1.7167 | 1.6578 | 1.7069 | 1.706742 | 0.036523 |
| 22 | 1.7101 | 1.6524 | 1.7038 | 1.702067 | 0.037087 |
| 22.5 | 1.7044 | 1.6433 | 1.6968 | 1.696117 | 0.039923 |
| 23 | 1.7016 | 1.6365 | 1.7027 | 1.6941 | 0.041514 |
| 23.5 | 1.688 | 1.6273 | 1.6845 | 1.681442 | 0.040686 |
| 24 | 1.6898 | 1.6332 | 1.6954 | 1.684825 | 0.036983 |

| **flgGi 37 °C** | | | | | |
| --- | --- | --- | --- | --- | --- |
| **Time (h)** | **1** | **2** | **3** | **Average** | **StDev** |
| 0 | 0.05 | 0.05 | 0.05 | 0.05 | 0 |
| 0.5 | 0.0893 | 0.0946 | 0.1187 | 0.098275 | 0.013804 |
| 1 | 0.1023 | 0.1021 | 0.1028 | 0.103267 | 0.001758 |
| 1.5 | 0.157 | 0.152 | 0.1569 | 0.15435 | 0.003009 |
| 2 | 0.2563 | 0.2537 | 0.2634 | 0.255175 | 0.006661 |
| 2.5 | 0.414 | 0.4141 | 0.4297 | 0.414958 | 0.011344 |
| 3 | 0.6674 | 0.6328 | 0.6417 | 0.638633 | 0.022708 |
| 3.5 | 0.9427 | 0.9213 | 0.9179 | 0.920483 | 0.017504 |
| 4 | 1.1276 | 1.1113 | 1.1174 | 1.104817 | 0.028699 |
| 4.5 | 1.2829 | 1.2729 | 1.269 | 1.252242 | 0.045759 |
| 5 | 1.4316 | 1.4311 | 1.4289 | 1.397917 | 0.065244 |
| 5.5 | 1.5315 | 1.536 | 1.5302 | 1.495658 | 0.073858 |
| 6 | 1.6229 | 1.629 | 1.6197 | 1.581433 | 0.084954 |
| 6.5 | 1.7008 | 1.7098 | 1.7002 | 1.656883 | 0.093536 |
| 7 | 1.782 | 1.8001 | 1.764 | 1.733917 | 0.097355 |
| 7.5 | 1.8254 | 1.8461 | 1.8329 | 1.810492 | 0.049364 |
| 8 | 1.8413 | 1.8599 | 1.8572 | 1.842092 | 0.022935 |
| 8.5 | 1.8446 | 1.8662 | 1.87 | 1.856108 | 0.013939 |
| 9 | 1.8352 | 1.8596 | 1.8692 | 1.854083 | 0.01436 |
| 9.5 | 1.832 | 1.854 | 1.864 | 1.851267 | 0.013605 |
| 10 | 1.832 | 1.8544 | 1.8616 | 1.851117 | 0.013099 |
| 10.5 | 1.8317 | 1.8535 | 1.8674 | 1.852367 | 0.014996 |
| 11 | 1.8164 | 1.8327 | 1.8527 | 1.8386 | 0.017535 |
| 11.5 | 1.8062 | 1.8202 | 1.8427 | 1.8307 | 0.021475 |
| 12 | 1.7998 | 1.8135 | 1.8458 | 1.826092 | 0.023137 |
| 12.5 | 1.7993 | 1.806 | 1.8462 | 1.822342 | 0.023153 |
| 13 | 1.7968 | 1.7994 | 1.8489 | 1.821542 | 0.027277 |
| 13.5 | 1.7817 | 1.7751 | 1.8356 | 1.805983 | 0.032007 |
| 14 | 1.7705 | 1.7486 | 1.8299 | 1.794525 | 0.041365 |
| 14.5 | 1.7744 | 1.7445 | 1.8281 | 1.791592 | 0.039232 |
| 15 | 1.7683 | 1.7247 | 1.8207 | 1.782367 | 0.045123 |
| 15.5 | 1.759 | 1.7136 | 1.8127 | 1.774267 | 0.047599 |
| 16 | 1.7552 | 1.6971 | 1.8138 | 1.768025 | 0.053951 |
| 16.5 | 1.7637 | 1.6909 | 1.8058 | 1.764 | 0.051928 |
| 17 | 1.7399 | 1.6642 | 1.794 | 1.747483 | 0.060894 |
| 17.5 | 1.7387 | 1.6603 | 1.7929 | 1.745517 | 0.062041 |
| 18 | 1.7304 | 1.6516 | 1.7779 | 1.734775 | 0.059918 |
| 18.5 | 1.7179 | 1.6365 | 1.7774 | 1.72695 | 0.066368 |
| 19 | 1.7149 | 1.6326 | 1.7744 | 1.722083 | 0.065225 |
| 19.5 | 1.7038 | 1.6277 | 1.7694 | 1.717475 | 0.067324 |
| 20 | 1.694 | 1.6188 | 1.7617 | 1.709717 | 0.068803 |
| 20.5 | 1.687 | 1.6122 | 1.7534 | 1.70235 | 0.068151 |
| 21 | 1.6815 | 1.6062 | 1.7505 | 1.697583 | 0.069247 |
| 21.5 | 1.6748 | 1.6013 | 1.7457 | 1.691842 | 0.068981 |
| 22 | 1.6712 | 1.6018 | 1.7428 | 1.689442 | 0.067379 |
| 22.5 | 1.6635 | 1.5917 | 1.7343 | 1.682367 | 0.069741 |
| 23 | 1.662 | 1.5939 | 1.7327 | 1.68105 | 0.067334 |
| 23.5 | 1.6501 | 1.5828 | 1.7249 | 1.670942 | 0.06866 |
| 24 | 1.6491 | 1.587 | 1.7211 | 1.669525 | 0.064619 |

| **flgIi 37 °C** | | | | | |
| --- | --- | --- | --- | --- | --- |
| **Time (h)** | **1** | **2** | **3** | **Average** | **StDev** |
| 0 | 0.05 | 0.05 | 0.05 | 0.05 | 0 |
| 0.5 | 0.1005 | 0.0949 | 0.0988 | 0.096175 | 0.004450749 |
| 1 | 0.0988 | 0.0994 | 0.1108 | 0.103717 | 0.005703897 |
| 1.5 | 0.141 | 0.1422 | 0.1746 | 0.152325 | 0.015573776 |
| 2 | 0.2348 | 0.2429 | 0.3002 | 0.2563 | 0.029721036 |
| 2.5 | 0.3908 | 0.4025 | 0.4583 | 0.413408 | 0.030412612 |
| 3 | 0.5963 | 0.6141 | 0.7067 | 0.632433 | 0.050164086 |
| 3.5 | 0.8768 | 0.8988 | 0.9679 | 0.910883 | 0.039481233 |
| 4 | 1.0943 | 1.1054 | 1.1506 | 1.103317 | 0.036283498 |
| 4.5 | 1.2397 | 1.2528 | 1.3 | 1.244167 | 0.047650976 |
| 5 | 1.4057 | 1.4184 | 1.4502 | 1.393592 | 0.065098628 |
| 5.5 | 1.512 | 1.5287 | 1.5536 | 1.494808 | 0.075217848 |
| 6 | 1.6107 | 1.6235 | 1.6377 | 1.581508 | 0.085629721 |
| 6.5 | 1.6981 | 1.7156 | 1.7182 | 1.662158 | 0.097360009 |
| 7 | 1.7767 | 1.7951 | 1.7903 | 1.737917 | 0.099206547 |
| 7.5 | 1.8459 | 1.8602 | 1.8422 | 1.821467 | 0.056469284 |
| 8 | 1.8628 | 1.8762 | 1.8665 | 1.853867 | 0.029807121 |
| 8.5 | 1.8901 | 1.8988 | 1.8739 | 1.876608 | 0.024284294 |
| 9 | 1.8838 | 1.8783 | 1.8728 | 1.871808 | 0.013738035 |
| 9.5 | 1.879 | 1.8789 | 1.8625 | 1.868867 | 0.012032271 |
| 10 | 1.8826 | 1.8706 | 1.872 | 1.870417 | 0.010732764 |
| 10.5 | 1.8814 | 1.8743 | 1.8702 | 1.870692 | 0.010312789 |
| 11 | 1.8649 | 1.8587 | 1.8605 | 1.859175 | 0.005098611 |
| 11.5 | 1.8551 | 1.8422 | 1.8522 | 1.8508 | 0.005854343 |
| 12 | 1.8534 | 1.8306 | 1.852 | 1.845317 | 0.010433759 |
| 12.5 | 1.8446 | 1.8211 | 1.8513 | 1.838717 | 0.012961781 |
| 13 | 1.844 | 1.8195 | 1.8588 | 1.840842 | 0.016206934 |
| 13.5 | 1.8265 | 1.7997 | 1.8474 | 1.826283 | 0.019834286 |
| 14 | 1.8158 | 1.7831 | 1.8357 | 1.815925 | 0.023396064 |
| 14.5 | 1.8116 | 1.7762 | 1.8304 | 1.809392 | 0.023433695 |
| 15 | 1.8083 | 1.7727 | 1.828 | 1.806192 | 0.023758653 |
| 15.5 | 1.7948 | 1.7534 | 1.8182 | 1.794542 | 0.029149733 |
| 16 | 1.7874 | 1.7353 | 1.8087 | 1.78435 | 0.034043355 |
| 16.5 | 1.7858 | 1.7394 | 1.7992 | 1.78 | 0.027652607 |
| 17 | 1.7666 | 1.7151 | 1.7965 | 1.767508 | 0.037325955 |
| 17.5 | 1.7662 | 1.707 | 1.7955 | 1.764717 | 0.040532826 |
| 18 | 1.7578 | 1.6983 | 1.7861 | 1.75535 | 0.039896073 |
| 18.5 | 1.7507 | 1.694 | 1.782 | 1.750675 | 0.040144188 |
| 19 | 1.7378 | 1.6847 | 1.7711 | 1.740008 | 0.03970248 |
| 19.5 | 1.7306 | 1.6792 | 1.7729 | 1.737925 | 0.043555281 |
| 20 | 1.7232 | 1.6726 | 1.7684 | 1.732142 | 0.044640344 |
| 20.5 | 1.7221 | 1.6648 | 1.7558 | 1.724875 | 0.043175099 |
| 21 | 1.716 | 1.6639 | 1.7565 | 1.722133 | 0.042855597 |
| 21.5 | 1.7106 | 1.6548 | 1.7531 | 1.716017 | 0.044815164 |
| 22 | 1.7092 | 1.6502 | 1.7444 | 1.711442 | 0.043869481 |
| 22.5 | 1.7041 | 1.6514 | 1.7443 | 1.709942 | 0.042985453 |
| 23 | 1.6986 | 1.6549 | 1.7388 | 1.706975 | 0.039218055 |
| 23.5 | 1.6859 | 1.6394 | 1.7299 | 1.695292 | 0.042232587 |
| 24 | 1.6915 | 1.6375 | 1.7198 | 1.692425 | 0.039063314 |

| **flgJi 37 °C** | | | | | |
| --- | --- | --- | --- | --- | --- |
| **Time (h)** | **1** | **2** | **3** | **Average** | **StDev** |
| 0 | 0.05 | 0.05 | 0.05 | 0.05 | 0 |
| 0.5 | 0.0853 | 0.0963 | 0.0986 | 0.092675 | 0.005982 |
| 1 | 0.0977 | 0.1055 | 0.1068 | 0.103967 | 0.004213 |
| 1.5 | 0.1396 | 0.1582 | 0.1569 | 0.15155 | 0.008478 |
| 2 | 0.2253 | 0.2666 | 0.2568 | 0.249 | 0.017656 |
| 2.5 | 0.3804 | 0.427 | 0.4227 | 0.408033 | 0.021404 |
| 3 | 0.5782 | 0.6719 | 0.6321 | 0.623708 | 0.0391 |
| 3.5 | 0.8424 | 0.947 | 0.9122 | 0.900408 | 0.043493 |
| 4 | 1.0639 | 1.1289 | 1.1161 | 1.092967 | 0.034502 |
| 4.5 | 1.2203 | 1.2844 | 1.2749 | 1.240942 | 0.047227 |
| 5 | 1.3807 | 1.4432 | 1.4353 | 1.389817 | 0.065971 |
| 5.5 | 1.4999 | 1.5489 | 1.5418 | 1.493883 | 0.075783 |
| 6 | 1.5985 | 1.6459 | 1.6411 | 1.584908 | 0.089748 |
| 6.5 | 1.6905 | 1.728 | 1.7228 | 1.664508 | 0.099903 |
| 7 | 1.7718 | 1.8135 | 1.8048 | 1.744917 | 0.105113 |
| 7.5 | 1.8463 | 1.8821 | 1.8682 | 1.833542 | 0.065658 |
| 8 | 1.8728 | 1.8931 | 1.8845 | 1.865092 | 0.03768 |
| 8.5 | 1.8991 | 1.9097 | 1.9045 | 1.889233 | 0.030706 |
| 9 | 1.9033 | 1.8955 | 1.8938 | 1.886233 | 0.022975 |
| 9.5 | 1.9164 | 1.8887 | 1.8955 | 1.888917 | 0.025459 |
| 10 | 1.9351 | 1.8837 | 1.8996 | 1.893717 | 0.032838 |
| 10.5 | 1.9376 | 1.8897 | 1.8961 | 1.895067 | 0.033157 |
| 11 | 1.9247 | 1.8717 | 1.8847 | 1.883425 | 0.030512 |
| 11.5 | 1.9191 | 1.8608 | 1.8763 | 1.877475 | 0.029311 |
| 12 | 1.9092 | 1.8559 | 1.8741 | 1.871117 | 0.028042 |
| 12.5 | 1.9114 | 1.8509 | 1.8755 | 1.868917 | 0.032336 |
| 13 | 1.9106 | 1.846 | 1.8765 | 1.868542 | 0.032121 |
| 13.5 | 1.8909 | 1.8317 | 1.8634 | 1.854383 | 0.028586 |
| 14 | 1.8861 | 1.8198 | 1.8556 | 1.84765 | 0.029784 |
| 14.5 | 1.8829 | 1.8266 | 1.8607 | 1.847392 | 0.029753 |
| 15 | 1.8779 | 1.8128 | 1.8528 | 1.839817 | 0.031237 |
| 15.5 | 1.8686 | 1.8043 | 1.8475 | 1.833042 | 0.030288 |
| 16 | 1.8616 | 1.7903 | 1.8401 | 1.8245 | 0.032309 |
| 16.5 | 1.8572 | 1.7916 | 1.845 | 1.82235 | 0.033609 |
| 17 | 1.8471 | 1.7663 | 1.8231 | 1.807083 | 0.035373 |
| 17.5 | 1.8453 | 1.7662 | 1.8298 | 1.807867 | 0.036202 |
| 18 | 1.8386 | 1.7568 | 1.8235 | 1.799525 | 0.038036 |
| 18.5 | 1.833 | 1.7428 | 1.8135 | 1.791325 | 0.040075 |
| 19 | 1.8259 | 1.7446 | 1.8113 | 1.787058 | 0.037967 |
| 19.5 | 1.8183 | 1.7337 | 1.8051 | 1.781525 | 0.038089 |
| 20 | 1.8123 | 1.7246 | 1.7972 | 1.774617 | 0.038888 |
| 20.5 | 1.8105 | 1.7157 | 1.7899 | 1.768225 | 0.041418 |
| 21 | 1.8063 | 1.7063 | 1.7932 | 1.764483 | 0.045134 |
| 21.5 | 1.7973 | 1.6992 | 1.7847 | 1.756692 | 0.044206 |
| 22 | 1.8006 | 1.6969 | 1.784 | 1.755867 | 0.046414 |
| 22.5 | 1.7905 | 1.6889 | 1.7779 | 1.749317 | 0.045646 |
| 23 | 1.7902 | 1.6916 | 1.778 | 1.74885 | 0.044769 |
| 23.5 | 1.7759 | 1.6718 | 1.7569 | 1.732642 | 0.045483 |
| 24 | 1.778 | 1.6807 | 1.7685 | 1.737025 | 0.0451 |
